# Identifying influential nodes based on the disassortativity and community structure of complex network

**DOI:** 10.1038/s41598-024-59071-x

**Published:** 2024-04-11

**Authors:** Zuxi Wang, Ruixiang Huang, Dian Yang, Yuqiang Peng, Boyun Zhou, Zhong Chen

**Affiliations:** 1 School of Artificial Intelligence and Automation, Huazhong University of Science and Technology, Wuhan, 430074 People’s Republic of China; 2 National Key Laboratory of Multispectral Information Intelligent Processing Technology, Wuhan, 430074 People’s Republic of China; 3 Key Laboratory of Image Information Processing and Intelligent Control, Ministry of Education of China, Wuhan, 430074 People’s Republic of China; 4 School of Information and Electronic Engineering, Zhejiang Gongshang University, Hangzhou, 310018 People’s Republic of China

**Keywords:** Complex networks, Statistical physics, thermodynamics and nonlinear dynamics

## Abstract

The complex networks exhibit significant heterogeneity in node connections, resulting in a few nodes playing critical roles in various scenarios, including decision-making, disease control, and population immunity. Therefore, accurately identifying these influential nodes that play crucial roles in networks is very important. Many methods have been proposed in different fields to solve this issue. This paper focuses on the different types of disassortativity existing in networks and innovatively introduces the concept of disassortativity of the node, namely, the inconsistency between the degree of a node and the degrees of its neighboring nodes, and proposes a measure of disassortativity of the node (DoN) by a step function. Furthermore, the paper analyzes and indicates that in many real-world network applications, such as online social networks, the influence of nodes within the network is often associated with disassortativity of the node and the community boundary structure of the network. Thus, the influential metric of node based on disassortativity and community structure (mDC) is proposed. Extensive experiments are conducted in synthetic and real networks, and the performance of the DoN and mDC is validated through network robustness experiments and immune experiment of disease infection. Experimental and analytical results demonstrate that compared to other state-of-the-art centrality measures, the proposed methods (DoN and mDC) exhibits superior identification performance and efficiency, particularly in non-disassortative networks and networks with clear community structures. Furthermore, we find that the DoN and mDC exhibit high stability to network noise and inaccuracies of the network data.

## Introduction

The concept of complex networks^1^ arises from various complex systems encountered in our daily lives, such as city road networks^2^, social networks^3^, disease transmission networks^4^, power grids^5^, and more. It can be said that complex networks are closely related to our lives. In the analysis and study of complex networks, research on network robustness and information dissemination has garnered significant attention from many researchers^6^. Among them, the exploration and discovery of influential nodes within networks can control the spread of information in networks^7^, assist road authorities in making better decisions, and quickly contain the spread of diseases^8^. Therefore, the exploration of influential nodes holds significant practical importance in the study of complex networks.

The relationship between the measurement of influential nodes and the topological characteristics of the network is a fundamental issue^9^. Classical measurement methods consider the influence of nodes based on the macroscopic network topology, and they can be roughly divided into three categories^10^: Degree centrality strategies based on local network information^1,11^. Centrality strategies based on global network information, including betweenness centrality^12,13^, closeness centrality^14,15^, and k-shell decomposition strategies^16^. The third category consists of hybrid methods, which integrate both local and global information of nodes. For example, Yang et al.^17^ proposed the AOGC method using a gravity model, which combines information such as network location, neighborhood based topological structure and shortest path to calculate the node mass and node looseness distance. Yu et al.^18^ in the process of identifying critical nodes in complex networks drew inspiration from convolutional neural networks in deep learning.

Community structure is an important attribute of network. Methods for detecting online communities include Louvain^19^ and label propagation^20^. Recently, Kamal et al.^21^ proposed a DSSC method to detect the community structure of the network by using deep learning, and the time complexity of this method is close to linear. Kamal et al.^22^ also further integrated the topological structure and attribute information of the network from the perspective of attribute graph clustering to enhance the clustering results, and proposed WSNMF method. However, classical centrality measurement methods often overlook the prevalent community structure in real networks when considering the influential nodes. Wen et al.^23^ have revealed that changes in the topology of networks can have important impact on the node centrality. Therefore, to uncover the impact of network community structure on the importance of network nodes, many researchers have started to investigate centrality measurement methods based on network community structure. Masuda^24^ introduced the Mod Centrality method, which coarsens the network using its community structure and quantifies the contribution of various bridge nodes to connections using the method of eigenvector centrality. However, this method tends to assign greater weight to bridge nodes while overlooking the role of hub nodes within communities, where bridge nodes refer to nodes that have edges connecting to other communities. Gupta et al.^25^ proposed the Comm Centrality method, which utilizes the strength of the network’s community structure to weight the edges of nodes within the community as well as outside the community. This method is effective in identifying hub nodes and bridge nodes in the network. However, it overlooks the impact of the size of the community in which a node resides on its importance. Tulu et al.^26^ introduced the Community-based Mediator method, which quantifies the influence of a node in the network based on the entropy of random walks between communities. It suggests that the more mixed the connections of a node are, the higher its centrality value in the network. Ghalmane et al.^27^ introduced the Community Hub-Bridge method, which weights nodes based on the size of network communities and the number of communities reachable within one hop. It takes into account both the community size and the impact of bridges between communities on node importance. However, its performance tends to decrease as the strength of the network community structure weakens. Subsequently, Ghalmane et al.^28^ extended classic centrality measures to modular networks, calculating the local importance of node’s classic centrality metrics within communities and their global importance across other communities. Recently, Magelinski et al.^29^ introduced the Modularity Vitality method. It calculates the changes in the network’s community modularity when each node is removed. If the removal of a node results in a significant decrease in the network’s community modularity, it indicates that the node is more important in the network. However, experimental results suggest that this method tends to favor bridge nodes in small communities while overlooking hub nodes in larger communities.

Many facts indicate that the influence of the node is not only affected by its neighboring nodes but also related to the community structure. In this paper, we first characterize and analyze the disassortativity property of nodes in the network, specifically, the presence of neighboring nodes with degrees smaller than the node itself. In blog network, each node in the network represents a blogger who establishes connections by following each other. Whether a blogger is influential in blog network often depends on whether their content is known by more fellow bloggers. However, bloggers with different numbers of followers often exhibit distinct social behaviors. Bloggers with fewer followers tend to be more proactive in following influential bloggers, whereas those with more followers are less likely to share the content of other bloggers or follow other bloggers themselves. The underlying motivation behind this asymmetric following behavior is that bloggers with fewer followers hope to enhance their influence in the social network by sharing content from more influential bloggers, such as videos and updates. However, this behavior of sharing contributes to giving more attention to the blogger whose content is being shared, thereby increasing their influence in the blog network. This asymmetric following behavior in the social network is reflected in the disassortativity of nodes. On the other hand, influential bloggers across fields tend to have greater influence than influential bloggers within a specific field. The division of fields on the social network often corresponds to the network’s community structure. Therefore, both the disassortativity of nodes and the network’s community structure are important factors influencing the centrality measures of network nodes. Based on the analysis above, a measure of node disassortativity(DoN) using a step function is introduced and further combines information about the network’s community structure to propose the influential metric of nodes based on disassortativity and community structure (mDC). Finally, network robustness experiments and immune experiment of disease infection are used to validate the effectiveness of the proposed method (DoN and mDC).

Meanwhile, considering the practical application of the algorithms proposed (DoN and mDC), especially for large-scale networks, it inevitably involves issues of complexity and computational overhead. This will pose challenges in real-time or resource-constrained environments. On one hand, in the real world, networks are often dynamically evolving, and the analysis of dynamic networks is commonly conducted using network snapshots. In dynamic networks, the extent of changes in the network’s topology can lead to significant variations in the influential nodes identified by algorithms, and whether the algorithm’s time complexity can adapt to the requirements of network snapshot intervals is a practical consideration in applications. On the other hand, networks in real-life scenarios are often affected by noise and data inaccuracies, which can lead to changes in network topology and bias in identifying influential nodes by algorithms. Therefore, whether the algorithms proposed in this paper can maintain stability in identifying influential nodes under the influence of noise is also a question that needs to be considered.

The main contributions of this paper are as follows:

1. This paper focuses on the different types of disassortativity existing in networks and innovatively introduces the concept of disassortativity of the node, and provides the measure of Disassortativity of the Node(DoN).
2. Observing and discovering the significant correlation between the disassortativity of nodes and the community boundary structure on impacting the influence of nodes. Furthermore, the influential metric of node based on Disassortativity and network Community structure (mDC) is presented.
3. Analyzing the performance of DoN and mDC by network robustness experiments and immune experiment of disease infection. Compared with the existing state-of-the-art centrality metrics, when attacking the influential nodes identified by DoN and mDC, the largest connected subgraph size of network and the network efficiency can decrease at a faster speed. Particularly in non-disassortative networks or networks with clear community structures, the mDC performs better in identifying new influential nodes that cannot be recognized by existing centrality metrics and DoN.
4. The time complexity of DoN is $O(n^2)$ (approaching that of degree centrality), while the time complexity of mDC is $O(n^2+nlogn+n)$. Although the efficiency of DoN is superior to that of mDC, and DoN performs better in identifying influential nodes compared to most existing state-of-the-art centrality metrics, the performance of mDC in identifying influential nodes is even better than that of DoN and the runtime of mDC is not high.

The rest of this paper is organized as follows: In Methods, an introduction is provided to existing centrality metrics and three evaluation criteria. In Proposed Methods, the concept of disassortativity of node(DoN) and its measurement are proposed. Furthermore, the influential metric of nodes based on disassortativity and community structure (mDC) is proposed. And the time complexity analysis of DoN and mDC is given. In Results, the stability analysis of DoN and mDC is given. And the effectiveness of the proposed method (DoN and mDC) is validated by network robustness experiments and immune experiment of disease infection. In Discussion, there is a conclusion and the direction of further research in the future.

## Methods

In this paper, we use *G*(Graph) to represent a complex network, *V*(Vertex) to denote the set of nodes in the complex network, and *E*(Edge) to represent the set of edges in the complex network. The expression for a complex network is defined as $G=\left( V,E\right)$. Let $n=\left| V\right|$ represent the number of network nodes, and $m=\left| E\right|$ represent the number of edges in the network. We represent the network structure in the form of an adjacency matrix, $A=\left[ a_{ij}\right] _{n\times n}$, where $a_{ij}\in R^n$. If there is an edge between node *i* and node *j*, $a_{ij}=1$; otherwise, $a_{ij}=0$.

### Network centrality measures

In order to identify influential nodes in complex networks, numerous researchers have proposed various centrality metrics from different perspectives. Among them, node degree, betweenness centrality, and closeness centrality are classical centrality metrics in network analysis, and these metrics are often used as benchmarks for comparing centrality. Additionally, recently introduced centrality metrics related to network community structure are also the subjects of comparison in this paper. Next, we will introduce them individually.

#### Degree centrality

Degree of node^1,11^ is a fundamental attribute of a node and is also the most intuitive criterion for assessing the importance of nodes in a network. The more edges a node has in the network, the more important it is considered to be in the network. Let $D_c\left( i\right)$ represent the degree centrality metric of node *i*.

1 $$\begin{aligned}{}&{D(i)=\frac{d_i}{n-1}}&\end{aligned}$$

where $d_i$ is the number of neighboring nodes of node *i*, and *n* represents the number of nodes in the network.

#### Betweenness centrality

In a connected complex network, there is always a shortest path from one node to another. Among all the shortest paths between pairs of nodes in the network, some nodes appear with particularly high frequency. Researchers consider such nodes as critical nodes in the network. Betweenness Centrality^12,13^ was proposed.

2 $$\begin{aligned}{}&{B(i)=\sum _{j,k\in V,i\ne j\ne k}\frac{l_{jk}\left( i\right) }{l_{jk}}\ }&\end{aligned}$$

where $l_{jk}$ represents the number of shortest paths from node *j* to node *k*, and $l_{jk}\left( i\right)$ represents the number of shortest paths from node *j* to node *k* that pass through node *i*.

#### Closeness centrality

Closeness Centrality^14,15^ eliminates the interference of outlier values by calculating the average of the shortest paths from a node to all other nodes in the network. The smaller the average distance a node has to all other nodes in the network, the larger its closeness centrality. Closeness centrality can be understood as a measure of a node’s importance based on the average dissemination time of information in the network.

3 $$\begin{aligned}{}&{CC\left( i\right) =\sum _{j=1,j\ne i}^{n}\frac{1}{d_{ji}}}&\end{aligned}$$

where $d_{ji}$ represents the length of the shortest path from node *j* to node *i*. When node *j* is not reachable from node *i*, $d_{ji}=\infty$, and $\frac{1}{d_{ji}}$ is defined as 0.

#### Modularity vitality centrality

Magelinski et al.^29^ calculated each node’s influence by using the marginal effect of removing nodes on network modularity.

4 $$\begin{aligned}{}&{MV(i)\ = Q(G)-Q(G \backslash \{i\}) }&\end{aligned}$$

where *G* represents the network, *Q*(*G*) represents the calculation of the modularity metric for the network, and $Q(G \backslash \{i\})$ represents the modularity metric for the network when node *i* is removed.

#### Community Hub-Bridge centrality

Ghalmane et al.^27^ weighted nodes based on the size of network communities and the number of communities reachable within one hop.

5 $$\begin{aligned}{}&{CHB\left( i\right) _{i\in C_k}=Card\left( C_k\right) \times k_i^{intra}\left( C_k\right) +\beta _{NNC}\left( i\right) \times k_i^{inter}\left( C_k\right) \ }&\end{aligned}$$

where $C_k$ represents the $k_{th}$ community in which node *i* is located, $Card\left( C_k\right)$ represents the size of the community to which node *i* belongs, $k_i^{intra}\left( C_k\right)$ represents the degree of node *i* within the community, and $k_i^{inter}\left( C_k\right)$ represents the degree of node *i* between communities.

#### Community-based mediator centrality

Tulu et al.^26^ quantified the importance of a node in the network by considering the entropy of a node within communities and between communities. They believed that the more diverse the connections of a node, the higher its centrality value.

6 $$\begin{aligned}{}&{CBM\left( i\right) =H_i\times \frac{d_i}{\sum _{i=1}^{N}d_i} }&\end{aligned}$$

where $H_i=\left[ -\sum \left( \rho _i^{intra}log\left( \rho _i^{intra}\right) \right) \right] +\left[ -\sum \left( \rho _i^{inter}log\left( \rho _i^{inter}\right) \right) \right]$, $\rho _i^{intra}$ represents the ratio of node *i*’s degree within the community to node *i*’s degree, and $\rho _i^{inter}$ represents the ratio of node *i*’s degree between communities to node *i*’s degree. $d_i$ represents the degree of node *i*.

#### Domirank centrality

Engsig et al.^30^ quantifies the dominance of the networks’ nodes in their respective neighborhoods, introducing a centrality metric, DomiRank, that integrates local and global topological information via a tunable parameter. From the networks’ structure and function perspective, nodes with high values of DomiRank highlight fragile neighborhoods whose integrity and functionality are highly dependent on those dominant nodes.

7 $$\begin{aligned}{}&{\frac{d\Gamma (t)}{dt}=\alpha A(\theta 1_{N \times 1} - \Gamma (t))-\beta \Gamma (t)}&\end{aligned}$$

where $A\in \mathbb {R}_{N \times N}$ is the adjacency matrix of the network and $\alpha ,\beta ,\theta \in \mathbb {R}^{+}: lim_{t \rightarrow \infty }\Gamma (t)=\Gamma \in \mathbb {R}_{N \times 1}$.

#### Extended degree and E-shell hierarchy decomposition

Liu et al.^31^ proposed an extended degree to improve the classical degree. And E -shell hierarchy decomposition is put forward for determining nodes’ position through the network’s hierarchical structure. Then, based on the combination of these two components, a hybrid characteristic centrality is proposed for evaluating the importance of nodes.

8 $$\begin{aligned}{}&{k^{ex}(u)=\delta \times k(u)+(1-\delta )\times \sum _{v\in \phi (u)}k(v)}&\end{aligned}$$

where the degree and 1-order neighbors of node are denoted as *k*(*u*) and $\phi (u)$, respectively. The extended degree of node *u* denoted by $k^{ex}(u)$. $\delta \in [0,1]$ is a weight which reflects the dependence of $k^{ex}(u)$ on *k*(*u*).

#### Vertex entanglement centrality

Huang et.al^32^ analyzed quantum entanglement and introduced vertex entanglement (VE), an entanglement-based metric capable of quantifying the perturbations caused by individual vertices on spectral entropy, residing at the intersection of quantum information and network science.

9 $$\begin{aligned}{}&{E_{\tau }(v) = S_{\tau }(G_v) - S_{\tau }(G) \approx \frac{2m\tau }{ln2(N-C)}(\frac{C_v}{Z_v} - \frac{C}{Z})+log_2\frac{Z-v}{Z}}&\end{aligned}$$

where $C_v$ is the number of connected components of $G_v$. Time $\tau$ serves as a tunable parameter in the computation of VE, which enables the study of the network response at micro, meso, and macroscales.*N* represents the number of nodes, *C* represents the number of network connected subgraphs, and *m* represents connected edges.

### Evaluation criteria

To assess whether the identification of influential nodes by network centrality metrics is effective, after quantifying the influence of nodes in the network, we will examine the effectiveness of centrality metrics in terms of their impact on the network’s topological structure^6^ and their influence on information dissemination in the network^33^. The evaluation methods employed are as follows:

#### Largest connected subgraph size

We use the Largest Connected Subgraph Size of the network to study the impact of nodes selected based on various centrality metrics on the overall network connectivity. When nodes in the network fail due to attacks, the initial network may be fragmented into multiple subnets. It is believed that a more intact network exhibits greater resilience when under attack. Therefore, the ratio of the size of the largest connected subgraph within the network to the size of the initial network is referred to as the largest Cconnected subgraph size (LCSS)^16^.

10 $$\begin{aligned}{}&{LCSS\left( G\right) =\frac{n_{max}}{n} }&\end{aligned}$$

where $n_{max}$ represents the number of nodes in the largest connected subgraph in the network, and *n* represents the size of the network. After being attacked, a smaller size of the largest connected subgraph indicates that the attacked nodes play more central role in the network.

#### Network efficiency

We employ network efficiency to investigate the impact of nodes selected based on various centrality metrics on the reachability between any two nodes in the network. Network efficiency^34^, denoted as NE, is a metric that quantifies the connectivity between nodes in a network. This metric posits that shorter shortest paths between nodes in the network lead to stronger network connectivity, better network performance, and is often used as a measure of network robustness. A higher network efficiency after network attacks indicates greater robustness.

11 $$\begin{aligned}{}&{NE\left( G\right) =\frac{1}{n\left( n-1\right) }\sum _{i\in V}\sum _{i\ne j,j\in V}{l_{ij}}^{-1} }&\end{aligned}$$

where *n* represents the total number of nodes in the network, $l_{ij}^{-1}$ represents the reciprocal of the shortest path from node *i* to node *j* in the network. If there is no path between these two nodes, $l_{ij}^{-1}$ is set to 0. After being attacked, a smaller network efficiency indicates the increased importance of the attacked node.

#### The information diffusion mechanism

We use the SIR model^35^ to study the effectiveness of nodes selected based on various centrality metrics in information dissemination in the network. The specific steps are as follows: In the initial state of the network, we designate the top *k* percent of nodes obtained from various centrality metrics as immune nodes. Then, among the remaining nodes, we randomly select one node as the infected node, while the rest of the nodes are considered susceptible nodes. In the network, let *S*(*t*) represents the number of susceptible nodes, *I*(*t*) represents the number of infected nodes, and *R*(*t*) represents the number of recovered nodes that cannot be infected again. The state changes of nodes in the network can be described using differential equations.

12 $$\begin{aligned}{}&{ \left\{ \begin{array}{lc} \frac{dS}{dt}=-\beta S\left( t\right) I\left( t\right) \\ \frac{dI}{dt}=\beta S\left( t\right) I\left( t\right) -\gamma I\left( t\right) \\ \frac{dR}{dt}=\gamma I\left( t\right) \end{array} \right. }&\end{aligned}$$

where $\beta$ represents the infection rate of network nodes, and $\gamma$ represents the recovery rate of network nodes. Specifically, at each time step, each infected node infects its susceptible neighbors with a probability of $\beta$, and then recovers from the disease with a probability of $\gamma$. In the experiments, we set $\gamma =0$, and repeated this process until there were no infected nodes left. Finally, to ensure the reliability of the results, all results are the averages of at least 500 independent experiments.

We use the proportion of infected nodes in the network to reflect the true impact of initial immune nodes on information dissemination in the network. A smaller proportion of infected nodes indicates a higher influence of the initially immune nodes on information dissemination in the network, playing a more crucial role in containing the spread of the disease.

## The proposed methods

### Disassortativity of node(DoN) and its measurement

**Figure 1:**
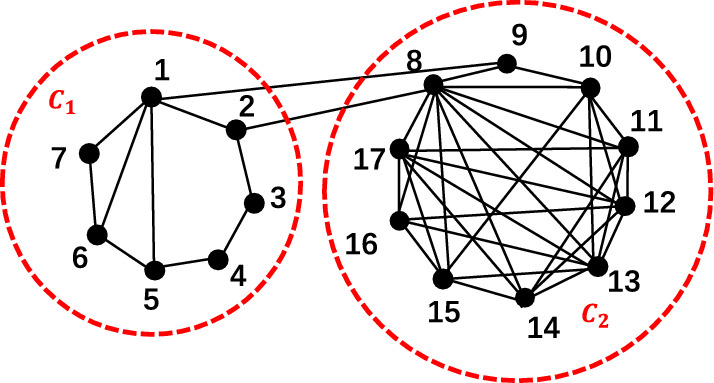
A toy network with 17 nodes and 38 edges. The network consists of two communities, respectively $C_1$ and $C_2$.

In the beginning, Newman et al.^36^ categorized networks into assortative networks, neutral networks, and disassortative networks to distinguish the connectivity preferences of nodes in the network. Among these, assortative networks refer to networks where high-degree nodes tend to connect with other high-degree nodes, meaning that the network exhibits a degree-degree positive correlation. Neutral networks, on the other hand, are networks where the presence of an edge between two nodes is unrelated to their degrees. Disassortative networks indicate networks where high-degree nodes tend to connect with low-degree nodes, implying a degree-degree negative correlation in the network. In this section, inspired by the phenomenon in disassortative networks where high-degree nodes tend to connect to low-degree nodes, we refer to the presence of neighbors with degrees smaller than the node’s own degree as the disassortativity of node. And node disassortativity is not limited to the assortative networks proposed by Newman, but exists in any network. Moreover, when a node has a greater number of neighbors with lower degrees, we refer to it as having a higher degree of disassortativity. In such cases, the node’s influence within the network is also greater. Just as in blog social networks, there exists an unequal following behavior among bloggers, This is because bloggers with a smaller number of followers are more likely to share content, which in turn leads to the blogger being followed by more users in the social network. Therefore, in social networks, the higher the disassortativity of a blogger, meaning being followed by more bloggers with fewer followers, the wider the reach of their work in the network, and the greater their influence as a blogger. In this analysis, we consider that truly important nodes in the network should have a greater influence on the network’s structure and functionality than their neighboring nodes. When a node’s neighboring nodes are more influential, the node itself becomes less likely to be influential.The measure of disassortativity of node(DoN) is given.

13 $$\begin{aligned}{}&{D{oN}_i=\sum _{j\in N_i} f\left( D_i-D_j\right) }&\end{aligned}$$

14 $$\begin{aligned}{}&{ f(D_i-D_j) = \left\{ \begin{array} {lr} 1 &{} D_i\ge D_j,j\in N_i \\ 0 &{} D_i<D_j,j\in N_i \end{array} \right. }&\end{aligned}$$

where $DoN_i$ represents the disassortativity measure for node *i*, $N_i$ denotes the set of neighbor nodes of node *i*, $D_i$ represents the degree of node *i*, and $D_j$ represents the degree of node *j*. It is worth noting that the range of $DoN_i$ is $\left[ 0,D_i\right]$, which cannot exceed the degree of node *i* itself. When $DoN_i=0$, it indicates that all neighboring nodes of node *i* have degrees greater than node *i* itself. Conversely, when $DoN_i=D_i$, it suggests that all neighboring nodes of node *i* have degrees smaller than node *i* itself, creating a star-like local structure within the first-order neighborhood. Moreover, the higher the disassortativity of node *i*, indicating higher influence. Conversely, a lower disassortativity measure suggests lower influence. We use a toy network to illustrate the properties of node disassortativity, as shown in Fig. 1. That is a toy network consisting of 17 nodes, with two communities, labeled as $C_1$ and $C_2$. Community $C_1$ comprises nodes labeled from 1 to 7, while community $C_2$ includes nodes labeled from 8 to 17. The degrees and disassortativity measures of all nodes in Fig. 1 are shown in Table 1.

**Figure 2:**
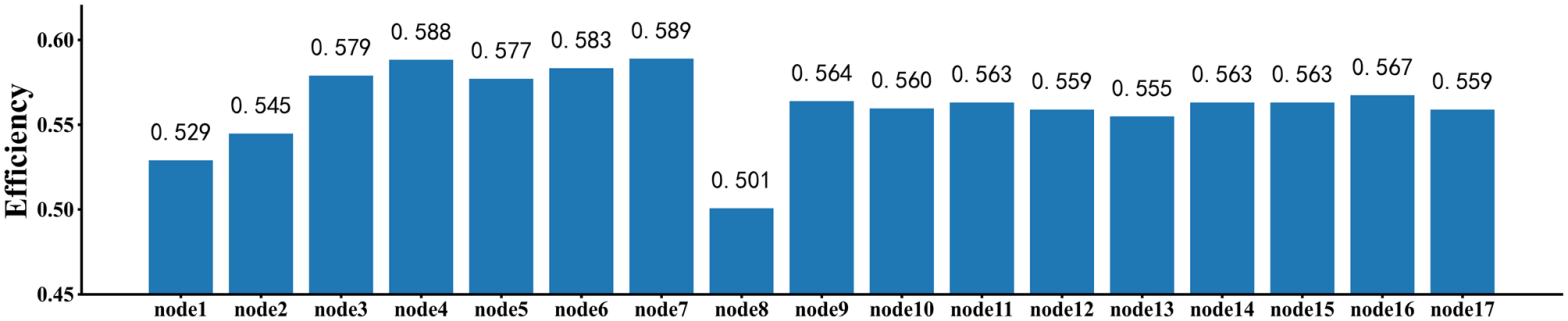
Bar chart of network efficiency. The horizontal axis represents the labels of nodes in the toy network, the vertical axis represents the network efficiency after removing the corresponding nodes. The lower the network efficiency, the more important the removal node is to the network performance.

To study the impact of each node in the toy network on network performance, we systematically removed each node depicted in Fig. 1 and analyzed the changes in network efficiency. The experimental results are shown in Fig. 2. A lower network efficiency after removing a node indicates a greater impact of the removed node on network connectivity, signifying its higher influence.

**Table 1:** The degrees and DoN of nodes in the toy network.

ID	Com	Degree	DoN	ID	Com	Degree	DoN
1	$C_1$	5	5	10	$C_2$	5	3
2	$C_1$	3	1	11	$C_2$	6	2
3	$C_1$	2	1	12	$C_2$	6	5
4	$C_1$	2	1	13	$C_2$	7	7
5	$C_1$	3	2	14	$C_2$	6	2
6	$C_1$	3	2	15	$C_2$	6	3
7	$C_1$	2	0	16	$C_2$	5	0
8	$C_2$	10	10	17	$C_2$	6	5
9	$C_2$	3	0				

In the table, ID represents the node’s label in the network, Com represents the community to which the node belongs, Degree represents the node’s degree, and DoN represents the disassortativity of node.

From Fig. 1, we can observe that nodes with high degrees do not necessarily have high disassortativity, as seen in the case of nodes 11 and 16. Node 11 has a degree of 6 but a disassortativity of only 2, while node 16 has a degree of 5 but an even lower disassortativity of 0. Additionally, when we consider both Figs. 1 and 2, we can see that nodes with the same degree can have different disassortativity measures. Furthermore, nodes with higher disassortativity tend to have greater influence. For instance, nodes 11 and 12 both have a degree of 6, but node 11 has a disassortativity of 2, while node 12 has a disassortativity of 5. According to Fig. 2, when node 12 is removed, the network efficiency is lower compared to when node 11 is removed. This suggests that removing node 12 has a greater impact on network connectivity, meaning that, for nodes with the same degree, node 12 has a higher influence than node 11.

The influence of nodes in the network is not only related to their individual disassortativity but also to the presence of community structures within the network. As shown in Fig. 1, node 2 has connections to community $C_2$. However, node 2 not only has a low degree but also a low disassortativity. Nonetheless, from Fig. 2, we can observe that when node 2 is removed, the network efficiency is much lower compared to the removal of nodes with higher disassortativity, such as node 13. Node 13 has a disassortativity of 7, but the network efficiency after removing node 13 is 0.5549, which is higher than the network efficiency of 0.5449 after removing node 2.Therefore, factors influencing the importance of network nodes are not only related to their individual disassortativity but also to the presence of community structures within the network.

### Influential metric based on node disassortativity and community structure(mDC)

In the real world, many studies^37–41^ have shown that most real networks exhibit a community structure, much like the domain-specific characteristics found in blog social networks. In blog social networks, we can observe that an influential blogger who spans multiple domains has a significantly higher level of influence and reach compared to bloggers who are influential only within a specific domain. The influence of each blogger depends not only on the number of their followers but also on whether they are a blogger spanning multiple domains and the number of regular bloggers interested in that domain. Based on the above analysis, in this section, we introduce an influential metric based on node disassortativity and community structure (mDC) by incorporating information about the network’s community structure. In large-scale complex networks, the presence of a community structure has a significant impact on information dissemination and network robustness. Communities within a network exhibit the following characteristics: the edges within a community are dense, while the edges between communities are sparse. We define the edge sets within a community and the edge sets between a community and other communities as $E_{C_i}^{in}$ and $E_{C_i}^{out}$.

15 $$\begin{aligned}{}&{E_{C_i}^{in}=\{l_{ij}\left| i,j\in V_{C_i}\right\} }&\end{aligned}$$

16 $$\begin{aligned}{}&{E_{C_i}^{out}=\{l_{ij}\left| i\in V_{C_i\ },j\notin V_{C_i}\right\} }&\end{aligned}$$

where $C_i$ represents community *i*, and $V_{C_i}$ represents the set of nodes within community *i*. Due to the presence of a community structure, we refer to nodes within a community that do not have edges connecting to other communities as ’internal nodes’, while nodes within a community that have edges connecting to other communities are called ’community boundary nodes’. The set of all boundary nodes within a community constitutes the boundary structure of that community. In particular, during the process of network information dissemination, the transmission of information between communities relies on the boundary structure of the communities. This means that information is disseminated from one community to another through the boundary nodes. When a community has fewer edges connecting it to other communities, the boundary nodes within that community play a more significant role in information dissemination. Additionally, the size of the communities connected also affects the importance of the boundary nodes; the larger the connected community, the wider the reach of information dissemination by the boundary nodes.Therefore, we consider that, in addition to node disassortativity, the community boundary structure of the network is also an important factor influencing the mDC centrality metric. Next, we will elaborate on how different aspects of the network’s community boundary structure impact the mDC centrality metric of nodes.

#### The community coefficient of a community

The community coefficient of a community is the ratio of the number of internal edges within a community to the total number of edges within that community. A higher community coefficient for a network community indicates stronger internal edge clustering, meaning the community has relatively fewer external edges connecting it with other communities. This also implies that the community can only exchange information with other communities through a limited number of external edges, emphasizing the importance of its boundary nodes in information dissemination. Next, we provide the formula for calculating the community coefficient of a network community.

17 $$\begin{aligned}{}&{\alpha _i=\frac{\left| E_{C_i}^{in}\right| }{\left| E_{C_i}^{in}\right| +\left| E_{C_i}^{out}\right| } }&\end{aligned}$$

where $C_i$ represents community *i*, and the formulas for calculating $E_{C_i}^{in}$ and $E_{C_i}^{out}$ are given by Eqs. (15) and (16), respectively, which represent the set of internal edges within community *i* and the set of edges connecting community *i* to other communities. $\left| E_{C_i}^{in}\right|$ and $\left| E_{C_i}^{out}\right|$ represent the number of elements in sets $E_{C_i}^{in}$ and $E_{C_i}^{out}$, respectively.

#### The community boundary popularity of a node

We define the community boundary popularity of a node as the number of the node connecting to other communities. The more connections a node has to other communities, and the larger the size of these connected communities, the higher the community boundary popularity of the node. Conversely, when a node is entirely within its own community, its boundary popularity is zero. In large-scale complex networks, community boundary nodes are associated with the interaction of a community with other communities in the network. In the process of information dissemination, if a community has a larger community coefficient, it indicates that the boundary nodes of that community are more influential for information dissemination. Additionally, if the community boundary nodes are connected to larger external communities, it implies that the information dissemination scope and influence of these boundary nodes are greater. Furthermore, a larger size of the node’s own community indicates that the boundary node has a stronger ability to receive information and has a greater influence within its own community.

18 $$\begin{aligned}{}&{ f_c(i) = \left\{ \begin{array} {lr} \sum _{j\in com}\alpha _i\times \left( 1+\frac{\left| C_i\right| +\left| C_j\right| }{2\left| C_{max}\right| }\right) &{} d_i\ne d_i^{in} \\ 0 &{} {\ d}_i=d_i^{in} \end{array} \right. }&\end{aligned}$$

19 $$\begin{aligned}{}&{d_i^{in}=\left| {j\ |j\in N_i, j\in V_{C_i}}\right| }&\end{aligned}$$

20 $$\begin{aligned}{}&{d_i^{out}=\left| {j\ |j\in N_i, j\notin V_{C_i}}\right| }&\end{aligned}$$

21 $$\begin{aligned}{}&{d_i=d_i^{in}+d_i^{out}\ }&\end{aligned}$$

where $d_i$ represents the degree of node *i*, $N_i$ represents the set of neighboring nodes of node *i*, $V_{C_i}$ represents the community to which the node belongs, $d_i^{in}$ represents the number of neighboring nodes that belong to the same community as node *i*, $d_i^{out}$ represents the number of neighboring nodes that do not belong to the same community as node *i*, and the relationship between $d_i$, $d_i^{in}$, and $d_i^{out}$ is given by Eq. (21). Additionally, com is the set of other communities connected to node *i*, $\alpha _i$ represents the community coefficient of node *i*’s belonging community, $\left| C_i\right|$ represents the number of nodes in the community to which node *i* belongs, $\left| C_j\right|$ represents the number of nodes in community *j*, and $\left| C_{max}\right|$ represents the number of nodes in the largest community.

#### The influential metric of node based on disassortativity and community structure(mDC)

Through analysis, the disassortativity of node and community boundary structure are both critical factors influencing the importance of nodes in the network. The higher the disassortativity of nodes in the network, the more obvious the community structure and the greater the popularity of nodes’ boundaries, the higher the influence of nodes. When the community structure is clear, the community boundary popularity of nodes plays a major role, while when the community structure is unclear, the node disassortativity plays a major role. Therefore, we use community coefficients to weighted sum the node disassortativity and the community boundary popularity.

22 $$\begin{aligned}{}&{mDC(i)=\left( 1-\alpha _i\right) \times DoN_i+\alpha _i\times f_c\left( i\right) }&\end{aligned}$$

where $\alpha _i$ is the community coefficient of node *i*, which is the attribute of network community. $DoN_i$ represents the node *i*’s disassortativity, and $f_c\left( i\right)$ represents the community boundary popularity of node *i*. $\alpha _i$ measures the degree of contact between the community where node i is located and other communities in the network.The larger $\alpha _i$ means that the community has less contact with the outside world, and the more important the nodes at the community boundary are, that is, the higher the weight of $f_c\left( i\right)$ in the mDC calculation formula. The algorithm of mDC is shown in algorithm 1. And the algorithm of DoN only needs to traverse the nodes of the network to obtain the degrees of neighbor nodes.

**Algorithm 1:**
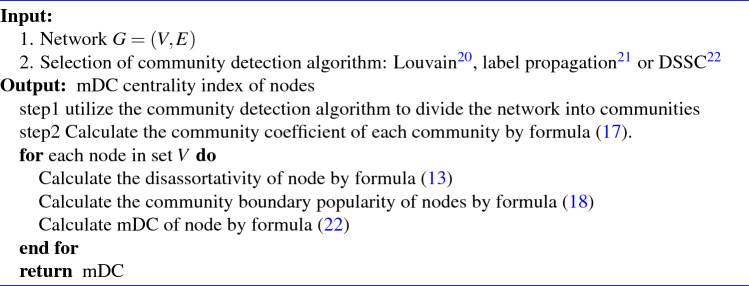
The Proposed mDC algorithm.

### Time complexity analysis of DoN and mDC

**Figure 3:**
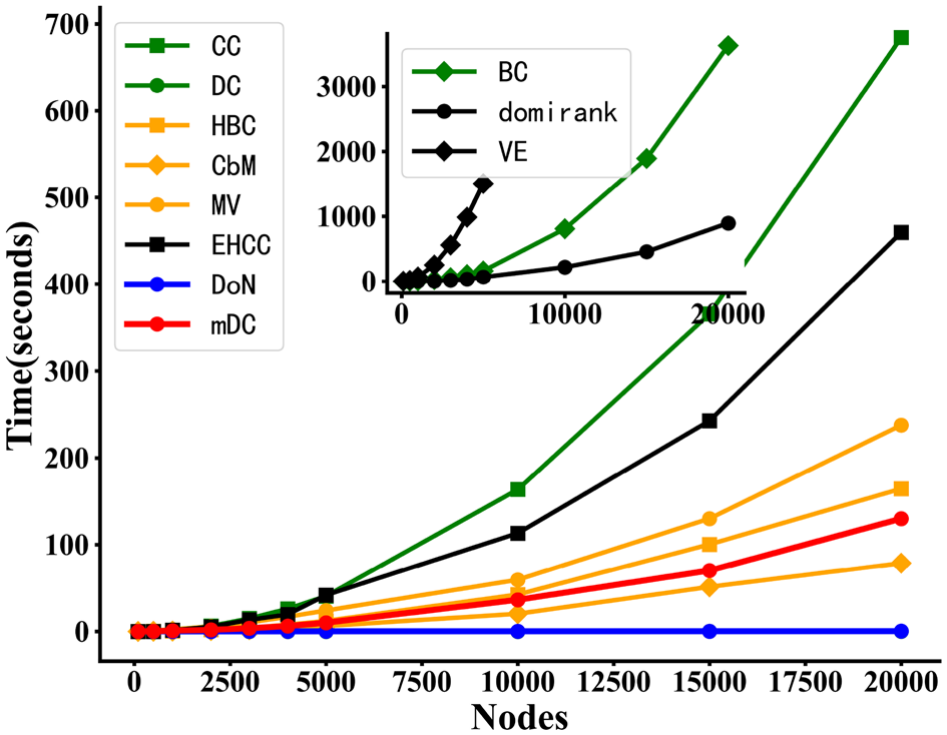
The comparison of the running time of DoN and mDC with other centrality metrics. The horizontal axis represents the network scale, while the vertical axis represents the runtime required for computing centrality metrics.

In this section, we provide an analysis of the time complexity of mDC algorithm. Understanding the time complexity of the algorithm helps to assess its efficiency and scalability. The time complexity of calculating disassortativity of node is $O(n^2)$. The time complexity of community coefficient depends on the time complexity of community detection algorithm. In this paper, we utilize Louvain community detection algorithm. The time complexity of the Louvain community detection algorithm is $O\left( nlogn\right)$. The time complexity of calculating the community boundary popularity of nodes is $O\left( n\right)$. Thus, the time complexity of mDC is $O\left( n^2+nlogn+n\right)$. On the other hand, the running time of DoN and mDC is compared with other centrality metrics, as shown in Fig. 3.

The analysis of the time complexity of the DoN and mDC centrality metrics will contribute to the application of the algorithms. For the application of large-scale network, we believe that the analysis, calculation and application of any algorithm have real-time challenges and resource-constrained settings. Although the time complexity of mDC algorithm is $O(n^2+nlogn+n)$, it is not computationally intensive. The measure of node disassortativity required by mDC does not need to be obtained through complicated calculation. The acquisition of community boundary structure information is related to the number of communities in the network. Compared with the number of nodes in the network, the number of communities will be lower than one order of magnitude, and the community boundary structure information does not involve complicated calculus. At present, the analysis of dynamic real-time network is usually through network snapshot analysis, and mDC can be suitable for applications whose operation time is less than the snapshot interval. If network snapshots in real life have strict timing requirements, we can use the faster-running DoN with a certain loss of accuracy. However, if timing is not a significant concern, we may consider using the mDC for better performance. The application scenarios of complex networks are problem-specific, and the proposed mDC can still meet the requirements of real-time and resource-constrained applications with relatively low demands.

In Fig. 3, the VE centrality metric cannot be computed for networks exceeding 5000 nodes due to memory constraints of the machine. Among them, the VE, EHCC and domirank centrality metrics are the latest proposed metrics. From Fig. 3, the computational efficiency of DoN is the highest (almost consistent with that of degree centrality(DC)). However, in the subsequent experiments, the performance of DoN is lower than that of mDC. For the mDC, we can find that the efficiency of mDC is higher compared to most centrality metrics. And the running time of mDC is much lower than the three centrality metrics recently proposed (VE, EHCC and domirank). In the subsequent experiments, the performance of the mDC in identifying influential nodes is the best.

## Results

To validate the effectiveness of the proposed DoN and mDC in identifying influential nodes in different networks, we conducted a series of experiments. Firstly, we analyzed the properties of DoN and mDC in networks with varying disassortativity and different community structure strengths. Futhermore, we analyzed the stability of DoN and mDC centrality metrics in response to dynamic network changes. Secondly, we designed robustness experiments based on network topology and simulated information dissemination experiments using the SIR model. Finally, through these experiments, we compared the performance of the proposed DoN and mDC with existing centrality metrics on synthetic networks of different sizes and eight real-world networks. The detailed experimental analyses are presented in the following sections.

### Dataset description and experimental environment

For synthetic networks, we generated networks using the LFR (Lancichinetti, Fortunato, and Radicchi) algorithm^42^, which produces networks with community sizes following a power-law distribution and degree distributions also following a power-law distribution. The algorithm’s parameters included $\gamma$ controlling the degree distribution exponent, $\beta$ controlling the power-law community size distribution, and $\mu$ as a mixing parameter controlling the strength of community structure within the synthetic network. The range for $\mu$ was set between [0, 1], where smaller values of $\mu$ indicated more pronounced community structures, higher modularity^38^. Table 2 provides the parameter values used for generating LFR networks, and Table 3 presents the topological characteristics of LFR synthetic networks of various sizes.

**Table 2:** The parameter settings for generating LFR synthetic networks, where $\gamma$ controls the degree distribution exponent of the synthetic network, $\beta$ controls the power-law distribution of community sizes, and $\mu$ regulates the strength of the network’s community structure.

Number of nodes *N*	500, 1000, 5000
Average degree $<k>$	10
Maximum degree $k_{max}$	180
Mixing parameter $\mu$	0.1, 0.8
$\gamma$	3
$\beta$	2
Minimum community size $C_{min}$	15
Maximum community size $C_{max}$	180

**Table 3:** The topological characteristics of different networks, where the naming convention for LFR synthetic networks follows the pattern LFR_N_$\mu$, where N represents the number of nodes in the network, $\mu$ represents the parameter controlling the strength of community structure in the synthetic network; *m* indicates the number of edges in the network; $k_{max}$ denotes the maximum degree in the network, $\mathrm {<}k\mathrm {>}$ represents the average degree of the network, $\mathrm {<C>}$ signifies the average clustering coefficient of the network^43^, *r* represents the degree assortativity of the network^44^; *M* denotes the modularity size of the network^38^, and the community detection algorithm used in this paper is the Louvain algorithm^19,45^; $\beta _{th}$ represents the disease propagation threshold of the network under the SIR model, calculated using the formula^46^ $\frac{<k>}{<k^2> <k>}$.

Networks	$\mu$	*N*	*m*	$k_{max}$	$\mathrm {<}k\mathrm {>}$	$\mathrm {<}C\mathrm {>}$	*r*	*M*	$\beta _{th}$
LFR500_0.1	0.1	500	2658	75	10.632	0.22	− 0.057	0.72	0.077
LFR500_0.8	0.8	500	2693	69	11.424	0.038	− 0.103	0.27	0.067
LFR1000_0.1	0.1	1000	2856	117	10.9	0.27	− 0.036	0.76	0.063
LFR1000_0.8	0.8	1000	5450	106	11.842	0.023	− 0.099	0.25	0.059
LFR5000_0.1	0.1	5000	28350	150	11.34	0.22	− 0.053	0.81	0.059
LFR5000_0.8	0.8	5000	28697	179	11.479	0.005	− 0.079	0.24	0.058
Email	–	1133	5451	71	9.622	0.22	0.078	0.56	0.054
PGP	–	10680	24316	205	4.554	0.27	0.238	0.88	0.056
Power	–	4941	6594	19	2.669	0.08	0.004	0.94	0.258
interactome_figeys	–	2239	6432	314	5.74	0.04	-0.330	0.47	0.018
collins_yeast	–	1622	9070	127	11.18	0.55	0.610	0.79	0.03
Webkb	–	348	16625	229	95.54	0.80	0.410	0.22	0.01
NS	–	1461	2742	34	3.76	0.69	0.46	0.96	0.17
new_zealand_collab	–	1511	4273	551	5.66	0.51	-0.33	0.46	0.01

In real network datasets, we employed the following datasets: Power representing the U.S. power grid network^43^, where each edge represents a power transmission line, and nodes represent generators, transformers, or substations. Email corresponds to the email communication network^47^, where nodes represent users, and each edge indicates at least one sent email. PGP network^48^ represents an interaction network of users of the PGP (Pretty Good Privacy) algorithm. Interactome_figeys^49^ denotes a network of human protein-protein interactions, with nodes representing proteins and edges representing interactions between two proteins. Collins_yeast^50^ represents a protein-protein interaction network in budding yeast (Saccharomyces cerevisiae). Webkb^51^ represents an interaction network among staff members in four computer science departments. NS^52^ stands for a collaboration network among scientists, where scientists sharing authorship on a paper are connected. new_zealand_collab^53^ is a network of scientific collaborations among institutions in New Zealand. Lastly, the topological characteristics of these actual networks are summarized in Table 3.

The comparison algorithms are implemented in Python and run on a PC with AMD Ryzen5 CPU of 2.10 GHz and 8 GB of RAM. The Package include python3.9.12, networkx2.8.4, numpy1.23.4, pandas1.5.1 and matplotlib3.5.1. And in the subsequent experiments, DC represents the degree centrality, BC represents the betweenness centrality, CC represents the closeness centrality, DoN stands for the node’s disassortativity proposed in this paper, mDC represents the node’s influence metric based on node disassortativity and community structure proposed in this paper, HBC represents the Community Hub-Bridge centrality, CbM represents the Community-based Mediator centrality, MV represents the Modularity Vitality centrality, EHCC represents Extended degree and Eshell hierarchy decomposition centrality, VE represents Vertex Entanglement centrality and dominrank represents Domirank centrality.

### Comparison of overlap of DoN and mDC with different disassortative networks

**Figure 4:**
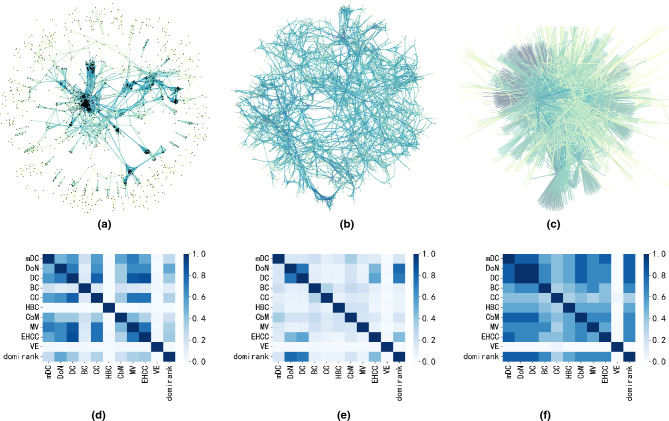
The overlap heatmap of the top 3% nodes between the proposed methiods (DoN and mDC) and other centrality metrics on three networks with different assortativity coefficients. Where r represents the assortativity coefficient of the network. Figure (**a**) is the collins_yeast network, an assortative network (r = 0.61 > 0), and Figure (**d**) is its overlap heatmap. Figure (**b**) is the power network, an neutral network (r = 0.004 $\approx$ 0), and Figure (**e**) is its overlap heatmap. Figure (**c**) is the interactome_figeys network, an disassortative network (r = − 0.33 < 0), and Figure (**f**) is its overlap heatmap. In the overlap heatmap, darker colors indicate a higher number of overlapping nodes among the top 3% identified by two centrality metrics, whereas lighter colors indicate a lower number of overlapping nodes. (**a**) collins_yeast r = 0.61. (**b**) power r = 0.004. (**c**) interactome_figeys r=-0.33. (**d**) heatmap of collins_yeast. (**e**) heatmap of power. (**f**) heatmap of interactome_figeys.

To investigate the properties of DoN and mDC in different disassortative networks, we selected three real networks with clear community structures but different assortativity coefficients^44^. The networks we selected are the collins_yeast network(assortative network), the power network(neutral network), and the interactome_figeys network(disassortative network). The collins_yeast network has an assortativity coefficient of 0.61 and a community modularity of 0.79, indicating that this network is assortative. The power network has an assortativity coefficient of 0.004 and a community modularity of almost 0, indicating that this network is degree-uncorrelated. The interactome_figeys network has an assortativity coefficient of -0.33 and a community modularity of 0.47, suggesting that this network is disassortative. This section further presents the overlap coefficient among the top 3% nodes identified by different centrality metrics in the three networks, as shown in Fig. 4d–f. The formula for calculating the overlap coefficient is as follows Eq. (23).

23 $$\begin{aligned}{}&{p=\frac{\left| A\cap B\right| }{\left| A\cup B\right| } }&\end{aligned}$$

where $|A \cap B|$ represents the number of elements in the intersection of sets A and B, and $|A \cap B|$ represents the number of elements in the union of sets A and B. In this section, A and B respectively represent the sets of the top 3% nodes identified by different centrality metrics.

From Fig. 4d, it can be observed that the overlap between the top 3% influential nodes identified by DoN and mDC is not very high. The mDC shows a high overlap coefficient DC and MV. However, there is low overlap coefficient between the nodes selected by mDC and those selected by BC. As indicated in Fig. 4a, in assortative networks, nodes tend to form rich-club phenomenon, with highly connected nodes preferentially linking to each other. This may be a contributing factor to the discrepancies in identifying influential nodes among different centrality metrics.

From Fig. 4e, we can find that the overlap coefficient between the top 3% nodes identified by mDC and different centrality metrics is almost close to zero, even the overlap coefficient between the influential nodes identified by other centrality metrics is almost close to zero. This may suggest that mDC has identified many influential nodes that were not identified by other centrality metrics. As indicated in Fig. 4b, in neutral networks, the connections between nodes are completely random. Different centrality metrics show significant differences in identifying influential nodes in the network.

From Fig. 4f , it reveals that in a disassortative network, such as Fig. 4c, there is a high overlap coefficient in the identification of influential nodes among different centrality metrics. As indicated in Fig. 4c, in disassortative networks, large-degree nodes tend to connect with small-degree nodes, creating a star-like structure. In such networks, large-degree nodes often act as hubs and connect to nodes with smaller degrees, making them easily identified as influential nodes by various centrality metrics, resulting in high overlap in different centrality metrics.

From the above analysis, it can be seen that different preferences for node connections in the network can produce biases in the identification of influential nodes by various centrality metrics. This is especially obvious for assortative networks and neutral networks, where the connections between nodes exhibit complexity. In these networks, nodes with high degrees are not necessarily the most influential nodes. However, DoN and mDC exhibits significant differences in identifying influential nodes in assortative and neutral networks compared to existing centrality metrics. The reason for this difference may be that DoN and mDC can identify disassortative subnetwork structures within assortative or neutral networks, which other centrality metrics cannot capture.

### Comparison of overlap of DoN and mDC with networks of different community structure

**Figure 5:**
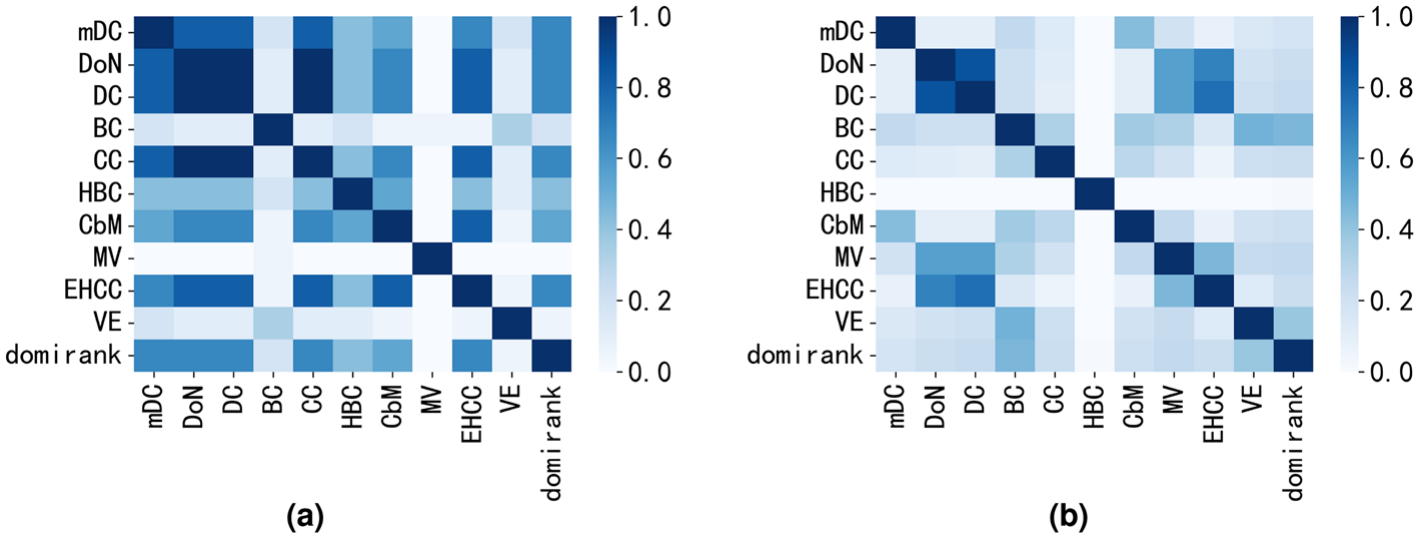
The overlap heatmap of the top 3% nodes between the proposed methods (DoN and mDC) and other centrality metrics on two networks with different community structure. Where r represents the assortativity coefficient of the network. Figure (**a**) is the overlap heatmap of webkb network(assortative network) with weak community structure and Figure (**b**) is the overlap heatmap of NS network(assortative network) with clear community structure. In the overlap heatmap, darker colors indicate a higher number of overlapping nodes among the top 3% identified by two centrality metrics, whereas lighter colors indicate a lower number of overlapping nodes. (**a**) webkb r = 0.41. (**b**) NS r = 0.46.

In this section, we will investigate the properties of DoN and mDC under different community structure strengths. We selected two real networks with similar assortativity coefficients but different community structure strengths. These two networks are the webkb network and the NS network. The webkb network has an assortativity coefficient of 0.41 and a community modularity of 0.22, indicating that the community structure in this network is not clear. The NS network has an assortativity coefficient of 0.46 and a community modularity of 0.96, indicating a more clear community structure with distinct boundaries between communities. We calculated the overlap coefficient among the top 3% nodes identified by each centrality metric using Eq. (23). The results are shown in Fig. 5.

The size of the network’s community modularity reflects the clarity of community boundaries. As evident from Fig. 5a, in networks where the community structure is not clear, the top 3% nodes selected by mDC exhibit high consistency with DoN, DC and CC but low consistency with BC and MV. And as seen in Fig. 5b, in networks with a clear community structure, the overlap coefficient between the top 3% nodes identified by mDC and different centrality metrics is almost close to zero, even the overlap coefficient between the influential nodes identified by other centrality metrics is almost close to zero.

From the above analysis, it suggests that DoN and mDC centrality can identify new influential nodes that other centrality metrics may not capture, particularly in networks with a clear community structure. This can be evidenced by the experimental results of subsequent robustness experiments and immune experiments of disease infection. And the above experimental analysis also demonstrates that the strength of network community structure has a significant impact on the identification of influential nodes.

### Stability analysis of DoN and mDC under network noise and inaccuracies

**Figure 6:**
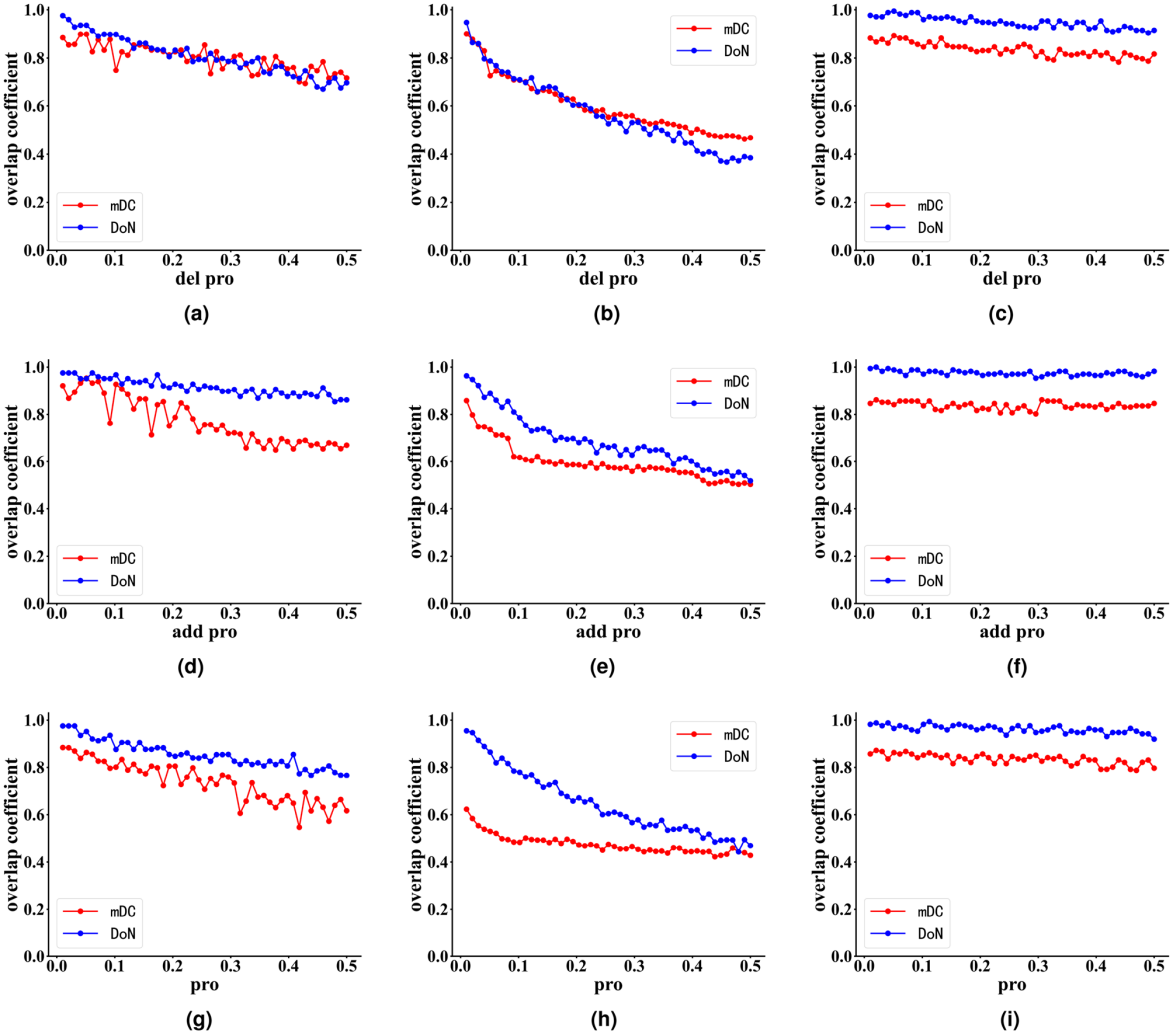
Stability Analysis of DoN and mDC under network noise and inaccuracies. Where the horizontal axis(start from 0.01) represents the proportion of dynamic changes in the number of edges in the network to the total number of edges in the original network, while the vertical axis represents the overlap coefficient between the DoN and mDC centrality metrics after network dynamic changes and before the changes. Figure (**a**) is the overlap curves of mDC and DoN by method (1) on collins_yeast network. Figure (**b**) is the overlap curves of mDC and DoN by method (1) on power network. Figure (**c**) is the overlap curves of mDC and DoN by method (1) on interactome_figeys network. Figure (**d**) is the overlap curves of mDC and DoN by method (2) on collins_yeast network. Figure (**e**) is the overlap curves of mDC and DoN by method (2) on power network. Figure (**f**) is the overlap curves of mDC and DoN by method (2) on interactome_figeys network. Figure (**g**) is the overlap curves of mDC and DoN by method (3) on collins_yeast network. Figure (**h**) is the overlap curves of mDC and DoN by method (3) on power network. Figure (**i**) is the overlap curves of mDC and DoN by method (3) on interactome_figeys network. The higher the curve in the Fig. 6, the more robust the centrality metric is to the dynamic changes of edges of network mentioned in this section. In other words, the centrality metric is more stable against noise and inaccuracies in the network. (**a**) overlap curves by (1) on collins_yeast. (**b**) overlap curves by (1) on power. (**c**) overlap curves by (1) on interactome_figeys. (**d**)  overlap curves by (2) on collins_yeast. (**e**) overlap curves by (2) on power. (**f**) overlap curves by (2) on interactome_figeys. (**g**) overlap curves by (3) on collins_yeast. (**h**) overlap curves by (3) on power. (**i**) overlap curves by (3) on interactome_figeys.

In real-world scenarios, network data may contain errors, missing edges, or noise, which can lead to misinterpretations of disassortativity and community structure. Such inaccuracies may result in the misidentification of influential nodes, leading to unreliable conclusions and recommendations. And the existence of network noise and inaccuracies may lead to the following three kinds of network edge changes, respectively, (1) randomly deleting edges with different proportions, (2) randomly adding edges with different proportions, and (3) there are both random deletions of edges and random additions of edges. To investigate the impact of the above three types of edge changes on the performance of DoN and mDC in identifying infuential nodes, we selected three different types of networks and subjected them to the above three types of edge changes to observe the variations in DoN and mDC. These three networks are respectively denoted as the collins_yeast network(assortative network), the power network(neutral network), and the interactome_figeys network(disassortative network). For different dynamic changes of each network, we calculate the overlap of the top 3% influential nodes before and after the network changes. The experimental results are shown in Fig. 6.

From Fig. 6a to c, it can be observed that in non-disassortative networks Fig. 6a and b, the DoN and mDC exhibit high stability against random edge deletions. In disassortative networks Fig. 6c, the DoN and mDC are also highly stability against random edge deletions. Even as the proportion of edge deletions in the network increases, the influential nodes identified by DoN and mDC do not undergo dramatic changes. The reason for this may be that the topology of disassortative networks generally exhibits a star-shaped structure, where hub nodes in the network tend to connect with low-degree nodes, and there is a significant disparity in degrees between them. Therefore, even after deleting a certain number of edges, the degree of hub nodes will still be much higher than that of low-degree nodes. Moreover, in disassortative networks, hub nodes always are the most influential nodes in the network.

From Fig.6d to f, it can be observed that in disassortative networks Fig. 6f, the DoN and mDC exhibit high stability against random edge additions. However, in assortative networks Fig. 6d, when the network undergoes random edge additions to a certain extent, both the DoN and mDC experience significant changes. Nevertheless, comparatively, the DoN tends to be more stable. In neutral networks Fig.6e, both the DoN and mDC show high sensitivity to random edge additions. When the scale of random edge additions reaches 50%, nearly 50% of the top 3% influential nodes in the network’s DoN and mDC also undergo changes.

From Fig. 6g to i, it can be observed that in disassortative networks Fig.6i, both the DoN and mDC exhibit high stability against random edge additions and deletions. In assortative networks Fig.6g, the stability trends of the DoN and mDC against random edge additions and deletions are similar. Nevertheless, comparatively, the DoN tends to be slightly more stable. In neutral networks Fig.6h, the mDC shows strong sensitivity to random edge additions and deletions, where adding or removing just 1% of the edges can cause changes in 40% of the top 3% influential nodes in the network. However, as the proportion of dynamic edge changes in the network increases, the changes in the top 3% influential nodes of mDC are not as dramatic.

From the analysis above, it is evident that the stability of the DoN and mDC varies across different types of network connectivity changes. They exhibit higher stability in disassortative networks, followed by assortative networks. In disassortative networks, the identification of the top 3% influential nodes by DoN and mDC does not undergo significant changes with the dynamic edge changes in the network. The reason for this may be that the topology of disassortative networks generally exhibits a star-shaped structure, where hub nodes in the network tend to connect with low-degree nodes, and there is a significant disparity in degrees between them. In neutral networks, the stability of DoN and mDC is the worst. However, in reality, it is rare for networks to undergo 50% edge changes in a short period of time. Therefore, for small-scale changes of edges, the influential nodes identified by DoN and mDC may not experience drastic variations. On the other hand, the experimental results mentioned above can guide us in updating the DoN and mDC centrality metrics according to the actual requirements of different types of network connectivity (assortative, neutral, disassortative network) when the network’s edge relationships undergo various degrees of change.

### Evaluation of DoN and mDC with network robustness experiment

The study of robustness in complex networks^6^ involves analyzing a network’s ability to withstand attacks. In this section, to validate the effectiveness of the DoN and mDC in identifying influential nodes that significantly impact a network’s topology and performance, we conducted robustness experiments on networks with different structures and compared them with existing state-of-the-art centrality metrics. We analyzed the size of the largest connected subgraph and network efficiency after subjecting the networks to attacks. A faster decline in the metrics indicates that the identified nodes are more influential to the network’s structure.

In robustness experiments, deliberate attacks were conducted on the network. Targeted attacks were performed by quantifying the influence of network nodes using existing centrality metrics and the centrality metric proposed in this paper. Nodes were ranked based on their centrality scores, and nodes with higher rankings were attacked first. The goal was to observe how the network’s performance declined at different proportions of important nodes being attacked. To ensure the reliability of the results, all results were based on the average of at least 500 independent experiments.

#### Robustness experiments in synthetic networks

**Figure 7:**
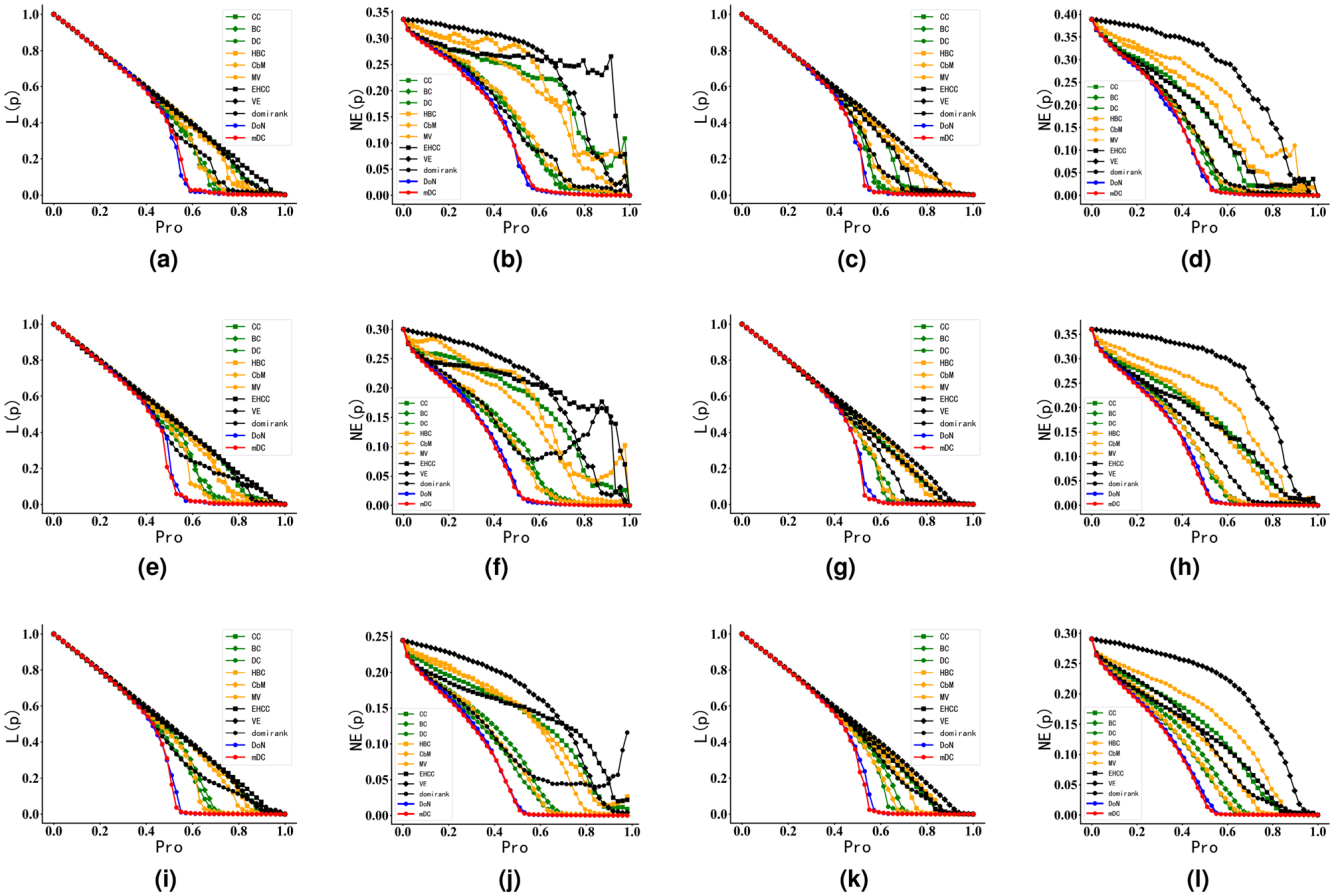
Robustness experiments of DoN and mDC on synthetic networks.The horizontal axis represents the proportion of nodes removed according to their influence. The L(p) of the ordinate represents the change of the network’s LCSS, and the NE(p) represents the change of network efficiency. The titles of the figures include “LFRnum”, where “num” signifies the number of nodes in the LFR synthetic network. $\mu$ is the parameter of LFR algorithm. The smaller the $\mu$, the more clear the community structure of the network. In Fig. 7, the faster the curve descends, the more influential attacked nodes are to the network topology and performance. (**a**) LFR500 μ = 0.1. (**b**) LFR500 μ = 0.1. (**c**) LFR500 μ = 0.8. (**d**) LFR500 μ = 0.8. (**e**) LFR1000 μ = 0.1. (**f**) LFR1000 μ = 0.1. (**g**) LFR1000 μ = 0.8. (**h**) LFR1000 μ = 0.8. (**i**) LFR5000 μ = 0.1. (**j**) LFR5000 μ = 0.1. (**k**) LFR5000 μ = 0.8. (**l**) LFR5000 μ = 0.8.

For LFR synthetic networks, we maintained the degree distribution exponent and the community size distribution exponent constant. We varied the mixing parameter, denoted as $\mu$, to generate synthetic networks with different levels of community structure strength.The topological characteristics of these synthetic networks are presented in Table 3. Finally, the results of the resilience experiments on synthetic networks with different community structure strengths are shown in Fig. 7.

The experimental results from Fig. 7 demonstrate that whether in networks with clear community structures or networks with unclear community structures, the disruption of influential nodes identified by the DoN and mDC metrics leads to a faster decline in both the network’s LCSS and network efficiency. Take the Fig. 7i from Fig. 7 as an example: when 50% of the nodes in mDC are disrupted, the LCSS in mDC has already dropped to nearly 0, whereas the LCSS in DoN still contains 10% of the nodes. In contrast, DC and BC still have almost 40% of the nodes in their maximum connected components. This indicates that the influential nodes identified by the mDC play a more influential role in shaping the network’s structure and performance compared to influential nodes identified by other existing centrality metrics. Furthermore, from the results of experiments with LFR5000 having two different community structure strengths, it can be observed that networks with stronger community structures reach the threshold of a LCSS dropping to 0 slightly earlier. This suggests that the identification performance of the mDC centrality metric is better in networks with more clear community structures than in networks where community structures are unclear.

From Fig. 7, it can be observed that for existing community-based centrality metrics like HBC, they tend to focus excessively on the size of network communities. This often results in the identification of influential nodes within larger communities. As a consequence, in the experiments, the nodes removed tend to be influential ones within large communities, while nodes within smaller communities remain intact, leading to the emergence of small-scale communities in the disrupted network. This phenomenon ultimately results in an unexpected increase in network efficiency, which was also observed in subsequent experiments with real networks. In contrast, due to its comprehensive consideration of network community structure information, the mDC exhibits more sTable and effective performance in identifying nodes crucial for network efficiency.

According to the experimental analysis above, it can be found that compared to existing centrality metrics, DoN and mDC can identify nodes that play a more influential role in the topology and performance of networks in LFR synthetic networks. Moreover, it performs better in identifying nodes in networks with clear community structures. On the other hand, compared to existing community-based centrality metrics, DoN-identified and mDC-identified influential nodes exhibit more stable effects on network efficiency changes.

#### Robustness experiments in real network

**Figure 8:**
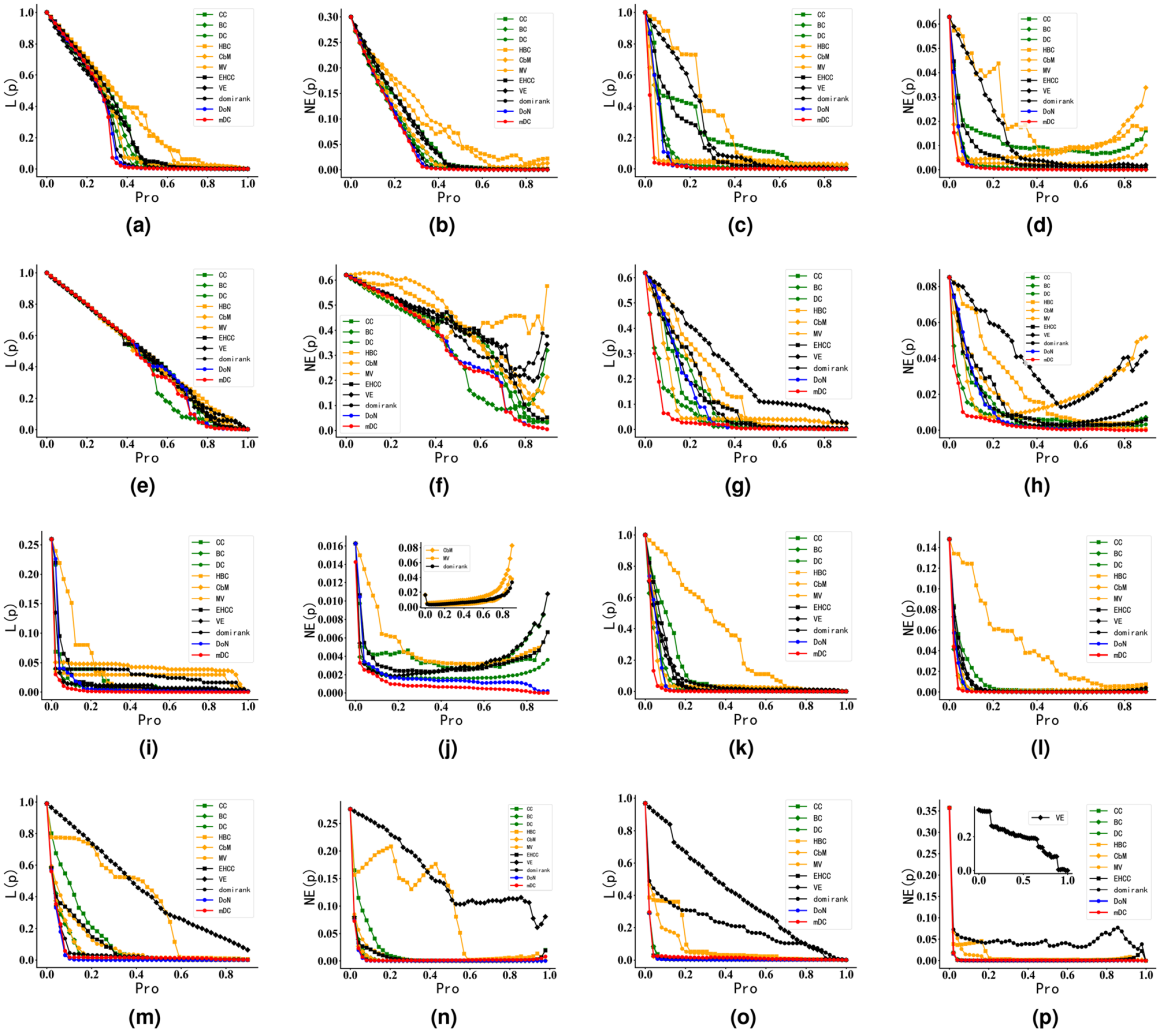
Robustness experiments of DoN and mDC on real networks. In Fig. 8, the horizontal axis represents the proportion of nodes destroyed, while the vertical axis represents the network’s performance changes. The L(p) of the ordinate represents the change of the network’s LCSS, and the NE(p) represents the change of network efficiency. Among them, webkb, PGP, collins_yeast, and NS belong to assortative networks, Email and Power networks are almost neutral networks, and interactome _figeys and new_zealand_collab networks are disassortative networks. In Fig. 8, the faster the curve descends, the more influential attacked nodes are to the network topology and performance. (**a**) Email. (**b**) Email. (**c**) power. (**d**) power. (**e**) webkb. (**f**) webkb. (**g**) collins_yeast. (**h**) collins_yeast. (**i**) NS. (**j**) NS. (**k**) PGP. (**l**) PGP. (**m**) interactome_figeys. (**n**) interactome_figeys. (**o**) new_zealand_collab. (**p**) new_zealand_collab.

This section presents robustness experiments for different centrality metrics conducted on real networks. The modularity sizes of different real networks are shown in Table 3. Among them, webkb, PGP, collins_yeast, and NS belong to assortative networks, Email and Power networks are almost neutral networks, and interactome_figeys and new_zealand_collab networks are disassortative networks. The results of robustness experiments on these real networks are depicted in Fig. 8.

From Fig. 8, in the assortative networks, such as the PGP (Fig. 8k,l) with an assortativity coefficient of 0.238, when only 4% of the network nodes identified by mDC are attacked, the network efficiency of the PGP has already dropped close to 0, and the LCSS has also decreased to around 3%. In contrast, traditional centrality metrics like DC and BC require disrupting 10% of the network’s nodes to achieve a similar effect. And Domirank, VE and EHCC centrality also need disrupt 20% of nodes to reach similar effect. For neutral networks, such as the Email (Fig. 8a,b) and Power (Fig. 8c,d), with assortativity coefficients of 0.078 and 0.004, respectively, disrupting nodes identified by the DoN and mDC leads to a faster decline in the network’s LCSS and network efficiency when compared to other centrality metrics, under the same proportion of nodes disrupted. Additionally, even in the case of disassortative networks like interactome_figeys (Fig. 8m,n) and new_zealand_collab (Fig. 8o,p), where the entire network exhibits a star-like structure with prominent influential nodes, the presence of some community structure in the network makes the proposed mDC more effective in identifying influential nodes than existing centrality metrics.

Furthermore, we can observe that existing community-based centrality metrics such as HBC, CbM, and even DC tend to prioritize hub nodes and bridge nodes within large-scale communities when identifying influential nodes influencing network efficiency. In NS network (Fig. 8j), we can find that except DoN and mDC, other centrality all lead to the increase of network efficiency. This phenomenon often overlooks influential nodes within smaller communities. Consequently, the disruption of hub nodes within large-scale communities often leads to the formation of numerous small-scale network fragments, causing network efficiency to increase as the proportion of disrupted nodes rises. This observation aligns with the results obtained from experiments on synthetic networks (Fig. 7f). It also highlights the stability and effectiveness of the DoN and mDC for identifying nodes influential to network efficiency.

The results in Fig. 8 indicate that regardless of the network’s preference for connecting certain types of nodes, the proposed DoN and mDC effectively identifies influential nodes within the network. On the other hand, it is evident that the mDC performs well in identifying influential nodes compared to existing centrality metrics and DoN. This is particularly notable in non-disassortative networks and networks with clear community structures, where the recognition performance of the mDC centrality metric is superior.

### Evaluation of DoN and mDC with susceptible-infected-removed

In this section, we further validated the performance of DoN and mDC in network disease propagation through SIR epidemic spreading experiments and compared them with existing state-of-the-art centrality metrics. The specific experimental method is as follows.

First, we immunize the top k% nodes obtained from each centrality metric, and then select a random node from the remaining set of nodes as initially infected node. We observe the proportion of infected nodes in the network after each iteration until there are no infected nodes left in the network. The smaller the proportion of infected nodes in the network, the higher the influence of the initially immunized nodes in promoting disease propagation in the network. This indicates a greater need for allocating additional resources to prevent the spread of disease in the context of disease control. In the experiments, we set the disease propagation probability $\beta$ to be $\alpha$ times the current network’s disease propagation threshold $\beta _{th}$, with $\alpha =0.5$ in subsequent experiments. Additionally, we set the node recovery rate $\gamma =0$ in order to record the final number of infected nodes in each experiment. Finally, to ensure the reliability of the results, all results are derived from the average of at least 500 independent epidemic spreading experiments.

#### SIR epidemic spreading experiments in synthetic networks

**Figure 9:**
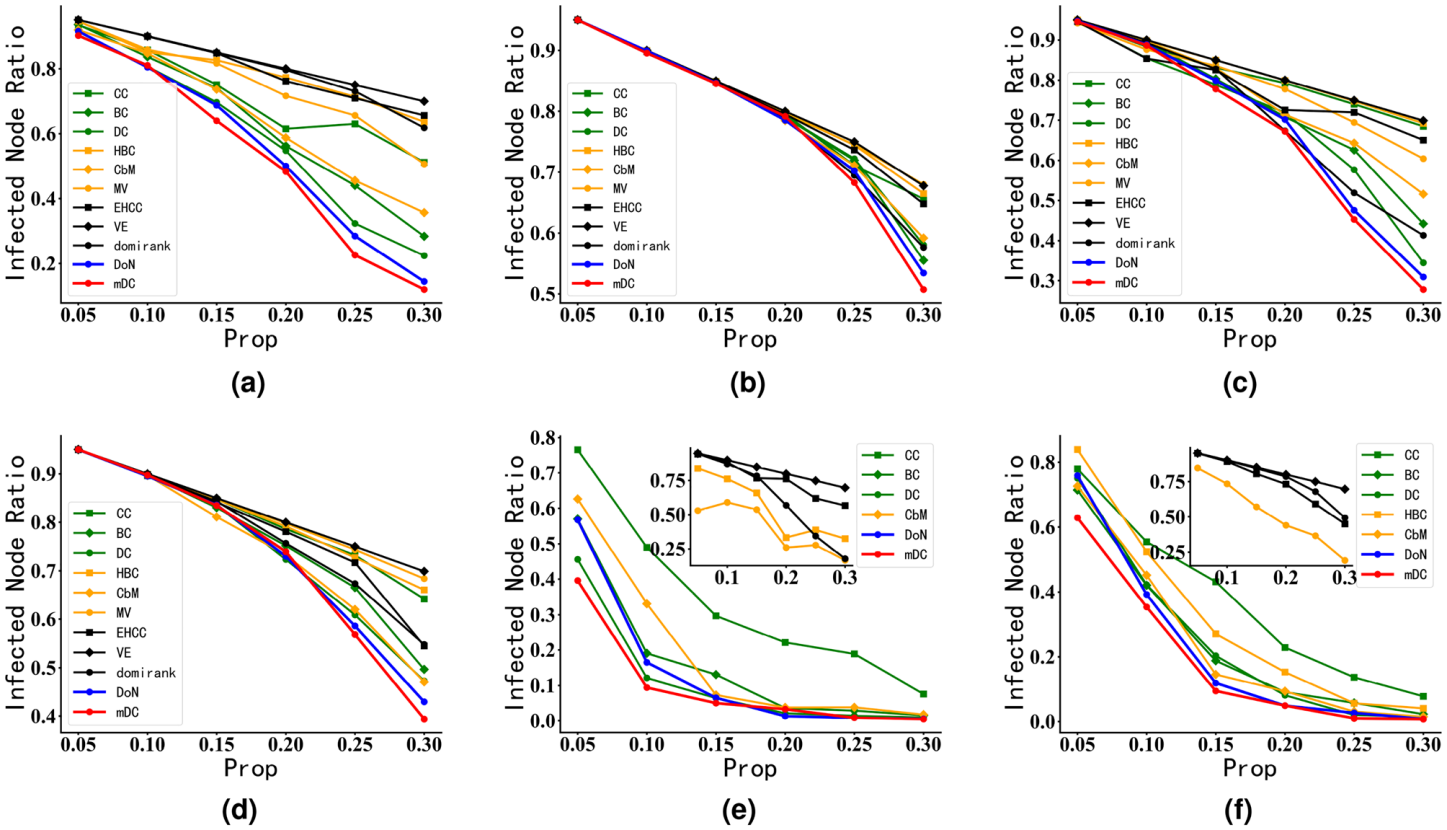
SIR epidemic spreading experiments of DoN and mDC in Synthetic Networks. The horizontal axis represents the proportion of immunized nodes in the network, while the vertical axis represents the ratio of nodes infected in the final network under the influence of the SIR disease spreading model. The titles of the figures include “LFRnum”, where “num” signifies the number of nodes in the LFR synthetic network. $\mu$ is the parameter of LFR algorithm. The smaller the $\mu$, the more clear the community structure of the network. Please see Table 3 for the relevant configuration of LFR network. In Fig. 9, the smaller the proportion of infected nodes in the network, the higher the influence of the initially immunized nodes in promoting information dissemination in the network. (**a**) LFR500 μ = 0.1. (**b**) LFR500 μ = 0.8. (**c**) LFR1000 μ = 0.1. (**d**) LFR1000 μ = 0.8. (**e**) LFR5000 μ = 0.1. (**f**) LFR5000 μ = 0.8.

During SIR epidemic spreading experiments conducted on synthetic networks of different scales, the disease propagation probability in the networks fluctuated within a small range around the respective disease propagation thresholds. This is because a high disease propagation probability can lead to rapid disease spread within the network, even if nodes identified are not high influential. Therefore, in the experiments, we set $\alpha =0.5$. Finally, the results of SIR epidemic spreading experiments on synthetic networks are shown in Fig. 9, where lower curves indicate that the initially immunized nodes are more influential in the network.

From the experimental results in Fig. 9, it can be observed that as the proportion of initially immunized nodes in the network increases, the proportion of infected nodes in the network decreases. However, compared to immunizing influential nodes identified by existing centrality measures, immunizing influential nodes identified by DoN and mDC leads to an even lower proportion of infected nodes in the network.This effect is particularly noticeable in networks with a clear community structure, such as the synthetic network Fig. 9e. For instance, immunizing 5% of nodes identified by mDC in the case of a clear community-structured network Fig. 9e results in 40% of nodes infected, while in the case of a network with unclear community structure Fig. 9f, the proportion of infected nodes is as high as 60%.

Furthermore, in the case of a clear community-structured LFR synthetic network such as Fig. 9e, immunizing the top 10% of nodes identified by mDC results in less than 10% of nodes being infected, and immunizing the top 10% of nodes identified by DoN results in about 15% of nodes being infected. In contrast, immunizing the top 10% of nodes identified by BC results in nearly 20% of nodes being infected. For EHCC and VE, immunizing the top 30% of nodes identified by them even leads to over 55% of nodes being infected. Immunizing the top 10% of nodes identified by HBC leads to almost 75% of nodes being infected. This indicates that immunizing these 10% of nodes is ineffective in preventing the disease spread. In other words, local immunization alone cannot achieve the same effect as global immunization, which may lead to a scenario in real life where a significant amount of resources is invested without effectively preventing the disease spread, resulting in substantial economic losses for society. Moreover, when compared to the remaining classical centrality, immunizing 10% of nodes leads to a higher number of infected nodes compared to immunizing nodes identified by mDC.

From the above analysis, it can be concluded that on synthetic networks, the DoN and mDC metric proposed in this paper is more effective in identifying influential nodes that have a significant impact on network information dissemination compared to existing centrality metrics. In networks of the same scale, the identification performance of the mDC is significantly better in networks with clear community structures compared to those with unclear community structures. The reason for this may lie in the fact that networks with clear community structures tend to have fewer edges between communities. Compared to other community-based centrality metrics, mDC can effectively consider boundary nodes between communities. In networks with unclear community structures, the identification performance of mDC may be somewhat lower due to unclear boundaries between network communities, but it still outperforms other centrality metrics.

#### SIR epidemic spreading experiments in real networks

**Figure 10:**
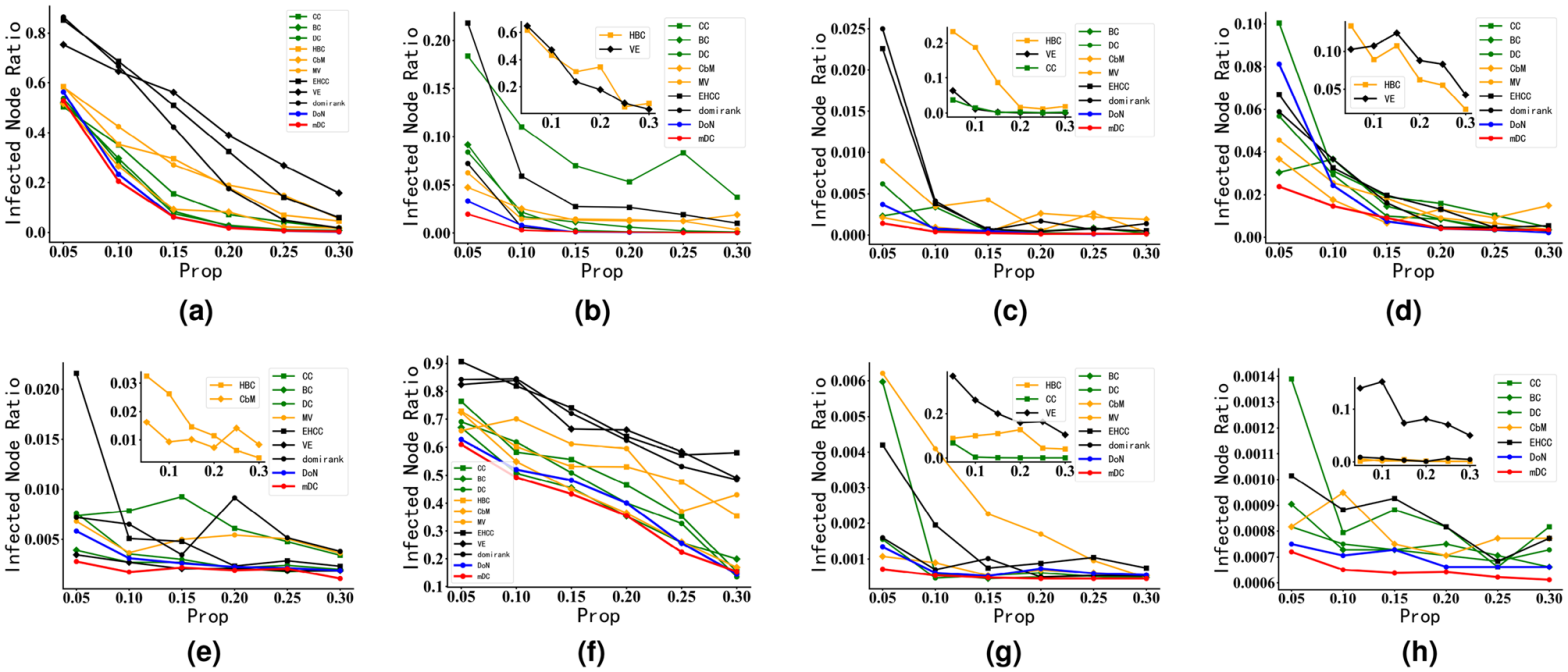
SIR epidemic spreading experiments of DoN and mDC in real networks. The horizontal axis represents the proportion of immunized nodes in each experiment, while the vertical axis represents the ratio of infected nodes in the final network under the SIR disease propagation model. Among them, webkb, PGP, collins_yeast, and NS belong to assortative networks, Email and Power networks are almost neutral networks, and interactome _figeys and new_zealand_collab networks are disassortative networks. In Fig. 10, the smaller the proportion of infected nodes in the network, the more influential the initially immunized nodes in promoting information dissemination in the network. (**a**) Email. (**b**) power. (**c**) PGP. (**d**) collins_yeast. (**e**) NS. (**f**) webkb. (**g**) interactome_figeys. (**h**) new_zealand_collab.

In this section, we conducted the SIR epidemic spreading experiments in the real network. The experimental results are shown in Fig. 10. Additionally, We record the top 5 of influential nodes selected by different centrality metrics in power network Fig. 10b shown in Table 5 and the proportion of infected nodes in the power network over the first 50 time steps shown in Table 4. A lower proportion of infected nodes in the network indicates a more influential role played by the initially immunized nodes in containing the epidemic spread throughout the network.

From the results in Fig. 10, it can be observed that whether in assortative or disassortative networks, at the same immunization ratio, immunizing the influential nodes identified by DoN and mDC can result in a lower proportion of infected nodes in the network. In the assortative network PGP (Fig. 10c), immunizing the initial 5% of nodes identified by mDC can keep the number of infected nodes in the network below 2% and the effect of immunizing the initial 5% of nodes identified by DoN is slightly worse. However, immunizing the top 5% of nodes identified by other centrality metrics would result in a higher number of infected nodes, especially the HBC, which leads to nearly 22% of nodes being infected. For neutral networks (Fig. 10d) and disassortative networks (Fig. 10f), immunizing the influential nodes identified by mDC also can result in a lower proportion of infected nodes than existing centrality metrics and DoN. This suggests that compared to existing centrality metrics, the mDC identifies influential nodes with a more central role in epidemic spreading.

**Table 4:** Infected proportion of initial immunized nodes in the power network for the top 5 nodes identified by different centrality metrics (1–50 time steps).

Node	1	2	3	4	5	10	20	30	40	50
1159	0.000202	0.000308	0.000474	0.000648	0.00085	0.003121	0.017677	0.053115	0.121688	0.22085
2312	0.000202	0.000324	0.000506	0.000708	0.001008	0.003679	0.020866	0.06342	0.135912	0.24034
**3468**	0.000202	0.000275	0.000393	0.000542	0.000777	0.002882	0.015471	0.047586	0.103967	**0.18606**
1166	0.000202	0.0003	0.000478	0.000793	0.001247	0.004408	0.023862	0.070868	0.150447	0.262635
2458	0.000202	0.000348	0.000478	0.000733	0.001004	0.00404	0.019907	0.06036	0.134394	0.236839
1308	0.000202	0.00032	0.000409	0.00053	0.000737	0.002603	0.016276	0.051451	0.110391	0.190427
1313	0.000202	0.000259	0.000348	0.00049	0.000704	0.003117	0.018353	0.053681	0.110557	0.196232
**2594**	0.000202	0.000385	0.000555	0.000785	0.001222	0.004444	0.017604	0.049702	0.10302	**0.185639**
**1442**	0.000202	0.000308	0.000474	0.00064	0.000838	0.003388	0.019008	0.053742	0.113131	**0.205991**
**2605**	0.000202	0.000312	0.000453	0.000615	0.000919	0.003165	0.018017	0.055041	0.122449	**0.224578**
2606	0.000202	0.000287	0.000409	0.000676	0.000967	0.003619	0.020919	0.06132	0.129532	0.221198
2553	0.000202	0.00038	0.000676	0.000895	0.001141	0.00459	0.024938	0.074131	0.156547	0.269358
2608	0.000202	0.000409	0.000611	0.000826	0.001044	0.00376	0.02098	0.065894	0.144829	0.253827
2607	0.000202	0.000372	0.000587	0.000814	0.00121	0.00427	0.021919	0.063222	0.13845	0.241562
2364	0.000202	0.00032	0.000518	0.000797	0.001166	0.004724	0.024687	0.069917	0.143853	0.24795
831	0.000202	0.000316	0.000474	0.00066	0.000866	0.00308	0.018519	0.061024	0.135118	0.234803
4164	0.000202	0.000332	0.000542	0.000708	0.000984	0.003805	0.020417	0.058069	0.120716	0.212212
1106	0.000202	0.000393	0.000591	0.00085	0.001214	0.004262	0.020757	0.062821	0.142259	0.247513
1243	0.000202	0.000332	0.000546	0.000769	0.001073	0.003586	0.016766	0.053216	0.116916	0.20986
2528	0.000202	0.000385	0.000575	0.000895	0.001235	0.004278	0.023311	0.064363	0.129136	0.223659
**4199**	0.000202	0.000328	0.000579	0.000866	0.001303	0.004392	0.023441	0.066335	0.140356	**0.235228**
4458	0.000202	0.000332	0.000534	0.000777	0.001146	0.004635	0.02595	0.072038	0.141554	0.243202
1131	0.000202	0.000352	0.000563	0.000866	0.001235	0.004023	0.022068	0.064586	0.131698	0.228852
2543	0.000202	0.000324	0.000571	0.000935	0.001368	0.004938	0.023862	0.065881	0.136159	0.225752
4345	0.000202	0.000308	0.000409	0.000579	0.000761	0.002825	0.015171	0.048946	0.11354	0.210917
2554	0.000202	0.000279	0.000389	0.00051	0.000716	0.002935	0.016794	0.054329	0.123546	0.231135
4219	0.000202	0.000348	0.00055	0.000866	0.001235	0.005335	0.027399	0.072504	0.136794	0.225238
4352	0.000202	0.000797	0.001833	0.003315	0.004986	0.0054070	0.021218	0.091188	0.15924	0.216397
4345	0.000203	0.0012102	0.002813	0.004906	0.00729	0.015531	0.050194	0.116833	0.174598	0.259128
615	0.0002023	0.0002712	0.0003804	0.000514	0.000737	0.003578	0.020959	0.055936	0.107638	0.201844
4458	0.0002024	0.000874	0.001704	0.002776	0.003867	0.014296	0.063205	0.15784	0.289884	0.435345
2668	0.0002023	0.000400	0.000907	0.001509	0.002348	0.009945	0.060781	0.136130	0.217409	0.353706
3468	0.000202	0.000696	0.001275	0.001765	0.002327	0.006237	0.025132	0.062270	0.115515	0.2276830
4110	0.000202	0.000502	0.000971	0.001679	0.002651	0.009710	0.042910	0.123671	0.244901	0.3726897
2382	0.0002023	0.000663	0.001149	0.001769	0.00249	0.008237	0.026513	0.063902	0.153058	0.336414
3537	0.000202	0.000279	0.000352	0.000421	0.000555	0.001125	0.005671	0.024359	0.132214	0.2117328
2553	0.0002023	0.001121	0.002198	0.003517	0.005027	0.018048	0.07355	0.148731	0.239696	0.385687
4381	0.0002023	0.00097	0.002489	0.004638	0.007047	0.015535	0.091748	0.0732321	0.158546	0.273260

The smaller the proportion of infected nodes at time 50, the more influential the initially immunized node is. Node represents the node label, and the bold portion represents the top 5 nodes identified by mDC in power network. The top 5 influential nodes identified by other centrality metrics in the power network are shown in Table 5.

In Table 4, we have listed the proportions of infected nodes in power network at each time step when these nodes are individually immunized as initial nodes. From Table 4, it can be observed that in the power network, node 3468 plays a crucial role in preventing disease spread. In Table 5, although both DC and mDC can identify node 3468, compared to DC, the mDC ranks node 3468 as the top node, while DC places it fourth and ranks node 2553 as the top node. However, experiments show that node 2553 is far less effective in preventing disease spread compared to node 3468. Furthermore, from Tables 4 and 5, we can conclude that nodes with higher degrees in the network are not necessarily the most effective at controlling information dissemination within the network. In fact, certain nodes with relatively lower degrees can be more effective at controlling information spread, such as node 2594 identified by the mDC, which is not among the top 5 nodes identified by DC.

**Table 5:** Top 5 influential nodes selected by different centrality metrics in power network.

Method	mDC	DC	BC	CC	DoN	HBC	CbM	MV	EHCC	VE	Domirank
1	3468	2553	4164	1308	2553	1106	4458	1166	2553	2668	2553
2	2594	4458	2543	2594	4458	1166	2617	1442	4345	4110	4458
3	1442	4345	1243	2605	4345	1178	1166	4474	4458	615	831
4	2605	3468	4219	1131	3468	1167	2608	4199	4381	3537	3468
5	4199	831	2528	2606	831	4219	1106	2607	4352	4458	2382

From the above analysis, we can find that in the study of epidemic spreading, discovering effective immunization strategies to place nodes in an immune state in order to prevent the spread of diseases is a highly significant and meaningful research area. And identifying influential nodes in the network with powerful information dissemination capabilities is a crucial step in discovering effective immunization strategies. This section has demonstrated through SIR epidemic spreading experiments that the mDC can identify influential nodes in the network with powerful information dissemination capabilities. Especially in non-disassortative networks and networks with clear community structures, its identification performance is superior. This provides valuable insights for the future development of more efficient and accurate immunization strategies.

## Discussion

In large-scale complex networks, identifying influential nodes by combining local and global information presents certain challenges. Firstly, this paper characterizes and analyzes the existence of disassortativity of the node in networks, namely the inconsistency between the degree of a node and the degrees of its neighboring nodes. The more neighbor nodes with smaller degrees there are, the greater the degree of disassortativity of a node. The paper provides a measure about the disassortativity of a node (DoN) by using the step function. Additionally, through an analysis of real blog networks, it is observed that the influence of bloggers is often related to the disassortativity of nodes and the community boundary structure in the network. Furthermore, combining the disassortativity of nodes and community structure, the influential metric of node based on disassortativity and community structure (mDC) is proposed, which is of significance for robustness of netwirk and network immunization against disease.

In both the synthetic network and real network robustness experiments, as well as immune experiment of disease infection, mDC not only effectively identifies community boundary nodes but also recognizes hub nodes within each community. Compared to state-of-the-art centrality metrics, the mDC more effectively identifies influential nodes in different networks. Meanwhile, for the DoN, although its performance is inferior to mDC, it is still much better than most state-of-the-art centrality metrics. Existing centrality metrics based on community structure(HBC, CbM, MV) often perform well in networks with clear community structures, but their performance weakens or even falls below that of classical centrality metrics(DC, BC, CC) in networks with unclear community structures and non-disassortative networks. On the contrary, in networks with unclear community structures and non-disassortative networks, the proposed DoN and mDC still keeps high identification performance compared to state-of-the-art centrality metrics. sThis indicates that in non-disassortative networks, the DoN and mDC can effectively identify new influential nodes that existing state-of-the-art centrality metrics cannot recognize, specifically those hidden within the disassortativity subnetworks of non-disassortative network. In terms of time complexity, the time complexity of DoN is $O(n^2)$ (approaching that of degree centrality), while the time complexity of mDC is $O(n^2+nlogn+n)$. Although the efficiency of DoN is superior to that of mDC, the performance of mDC in identifying influential nodes is the best and the runtime of mDC is not high.

In the future, we will further consider: (1) We will consider non-overlapping community division for the division of network community structure, and further discuss how to effectively identify the influentia nodes in overlapping community structure; (2) The proposed DoN and mDC algorithm runs on the topology of the original network, and the popular network representation also has hyperbolic representation. In the future, we can further extend DoN and mDC algorithm to hyperbolic space of the network. (3) Our algorithm is mainly applied to static networks, while most real networks evolve dynamically. We will try to determine the influential nodes of the network snapshot at current moment through the influential nodes of the network snapshot at the previous moment and the network difference information between the network snapshot at current moment and the network snapshot at the previous moment to reduce the time complexity and repeated calculation of the algorithm in the dynamic network.

## Data Availability

Network data that being used in this article can be downloaded freely from the publicly accessible repositories https://github.c om/Hx4869/mDC-datasets.
